# Standardisation of flow cytometry for whole blood immunophenotyping of islet transplant and transplant clinical trial recipients

**DOI:** 10.1371/journal.pone.0217163

**Published:** 2019-05-22

**Authors:** Elvira Jimenez Vera, Yi Vee Chew, Leigh Nicholson, Heather Burns, Patricia Anderson, Hsiao-Ting Chen, Lindy Williams, Karen Keung, Negar Talaei Zanjani, Suat Dervish, Ellis Patrick, Xin Maggie Wang, Shounan Yi, Wayne Hawthorne, Stephen Alexander, Philip J. O’Connell, Min Hu

**Affiliations:** 1 Centre for Transplant and Renal Research, Westmead Institute for Medical Research, Sydney, Australia; 2 Flow Cytometry Core Facility, Westmead Institute for Medical Research, Sydney, Australia; 3 School of Mathematics and Statistics, The University of Sydney, Sydney, Australia; 4 Westmead Clinical Schools, Faculty of Medicine and Health, The University of Sydney, Sydney, Australia; 5 Centre for Kidney Research, Children's Hospital at Westmead, Sydney, Australia; Universite Paris-Sud, FRANCE

## Abstract

Understanding the immunological phenotype of transplant recipients is important to improve outcomes and develop new therapies. Immunophenotyping of whole peripheral blood (WPB) by flow cytometry is a rapid method to obtain large amounts of data relating to the outcomes of different transplant treatments with limited patient impact. Healthy individuals and patients with type 1 diabetes (T1D) enrolled in islet transplantation were recruited and WPB was collected. 46 fluorochrome-conjugated mouse-anti-human antibodies were used (43 of 46 antibodies were titrated). BD cytometer setup and tracking beads were used to characterize and adjust for cytometer performance. Antibody cocktails were pre-mixed <60 minutes before staining. Multicolour panels were designed based on fluorochrome brightness, antigen density, co-expression, and fluorochrome spillover into non-primary detectors in each panel on a 5 laser flow cytometer. WPB sample staining used 50–300 μl WPB for each panel and was performed within 2 hours of blood sample collection. Samples were acquired on a BD-LSRFortessa. The operating procedures, including specimen collection, antibody cocktails, staining protocol, flow-cytometer setup and data analysis, were standardized. The staining index of 43 antibodies and the spillover spreading matrix for each panel was calculated. The final concentrations for the 46 antibodies used was determined for staining of WPB samples. Absolute cell-count and 7 leukocyte profiling panels consisting of subsets and/or status of granulocytes, monocytes, dendritic, B, NK, and T cells including regulatory T cells (Tregs) and NKT were designed and established on a 5 laser BD-LSR Fortessa. 13 T1D patients, including 4 islet transplant recipients and 8 healthy controls, were evaluated. The ability to reproducibly measure immune subsets and immune-profiles of islet transplant patients up to 18 months post transplantation has been established as a tool to measure immune cell reconstitution after transplantation.

## Introduction

Understanding the immunological phenotype of transplant recipients on standard immunosuppression, immune-cell therapies and new drug treatments is essential for improving transplantation outcome. Immune monitoring by multi-colour flow cytometry is a highly useful tool to assess the immune response in transplantation, and has the potential to shed insight on the mechanisms responsible for successful treatment. Additionally, immunophenotyping of whole-peripheral-blood (WPB) by flow cytometry is a reliable, fast, and easy method to obtain large amounts of information on healthy individuals [1], on the effects and outcomes of different treatments in transplantation[2], and in the diagnosis of Leukemia and Lymphoma [3], with minimal impact on patients.

While short term outcomes after transplantation are excellent, long term outcomes remain unchanged despite the introduction of calcineurin inhibitors and newer anti-proliferative agents [4]. Transplant rejection remains a major cause of graft loss, and loss of function, with 18% of first grafts and 23.5% of subsequent grafts undergoing rejection in the first 6 months [5]. Acute rejection is a complex process resulting from activation of the adaptive immune response [6], which is initiated by activation of the innate immune system involving NK cells [7], monocytes-macrophages [8, 9] and neutrophils [10], that are involved in the initial steps of allorecognition. In adaptive immunity, innate immune recognition leads to T cell activation that plays a central role in the rejection process [11]. Additionally, FOXP3+ regulatory T cells (Tregs) are one of several types of regulatory cells that have been shown to protect against rejection and promote transplant tolerance in animal models and in clinical settings [12–17]. In the ‘ONE study’, a multicentre Phase I/II study of immune-cell therapies in kidney transplantation, naturally occurring FOXP3+Treg cells have been investigated for their safety and ability to prevent acute rejection after kidney transplantation (www.onestudy.org) [18, 19]. Apart from the role of well described T cell subsets, the role of B cells, particularly regulatory B cells (Bregs) [20], as well as dendritic cells (DCs) which are capable of promoting rejection or tolerance in transplantation, have also been studied [21, 22]. These studies have led to an increased awareness of the role of these immune subsets in both the initiation of rejection and the maintenance of non-responsiveness, especially in the clinical setting where often there is a period of acute short-term induction immunosuppressive therapy followed by a period of maintenance immunosuppression. Therefore, it has been proposed that immunophenotyping of patients may help identify a risk profile for acute rejection and/or graft loss.

The ‘One Study’ established 6 leukocyte profiling panels for monitoring the major immune cells involved in transplantation [2]. It was proposed as a standardized method for transplant patients involved in clinical trials and the method was optimized for use on a 10 colour, 3 laser, Navios flow cytometer (Beckman Coulter) [2]. The complexity of flow cytometry assays includes multi-step, multi-reagent assays followed by sample acquisition on multi-colour flow cytometers. Compatibility, applicability and standardization of staining panels across instrumentation is essential for results to be comparable and the methods to gain widespread acceptance. In this study, the aim was to build upon the “One Study” standardisation effort and develop a versatile but consistent flow cytometry method, involving the standardization of sample handling, reagents, antibodies cocktails, staining protocol, instrument setup and data analysis on a different platform for use in the monitoring of transplant patients. In this study, samples were analyzed on the BD-LSR Fortessa (BD Biosciences) in order to determine if the panels described for the ‘ONE Study’ could be acquired on other platforms, utilising additional lasers and UV excitable fluorochromes, identifying identical population subsets and determining the suitability for monitoring patients after pancreatic islet transplantation. Using only 1.5ml of WPB, we established absolute cell-count and 7 leukocyte-profiling panels, that identified subsets and/or states of B, NK, monocytes, DCs and granulocytes, while focusing on T cells including NKT and FOXP3+Tregs, on a 5 laser,18 parameter instrument. This method was found to be a reproducible and highly useful tool for monitoring changes in the immune-profile of individuals after islet transplantation and has the potential to be applied to a range of clinical trials and be used as a reference for higher parameter panel designs.

## Materials and methods

### Blood specimen collection

Healthy volunteers at the Westmead Institute for Medical Research, Westmead, NSW, Australia, and patients with T1D at Westmead Hospital who were enrolled in a trial of pancreatic islet transplantation were recruited to the study. The study was approved by the Human Research Ethics Committee of Western Sydney Local Health District. WPB was collected by one nominated nurse coordinator and one renal specialist. WPB was collected into 8ml EDTA vacutainers (BD Biosciences) and kept at room temperature (22°C to 30°C) [23]. 1.5ml of WPB was used in the study and 6.5ml of WPB was stored for later study.

### Antibodies and titration

46 fluorochrome-conjugated anti-human antibodies were used for the finalised panels (Table 1). All antibodies were supplied by BD Biosciences, except CD303 and CCR7 (CD197), which were obtained from Miltenyi Biotec and R&D Systems respectively. An additional 21 antibodies were tested for certain antigens of multiple clones and/or fluorochrome, listed in S1 Table. Antibody clones were chosen based on published results [2, 24] and from the results of our study. The 43 individual antibodies were titrated using anticoagulated-WPB of healthy-control samples under 5–6 serial dilutions of antibodies (1, 1/2, 1/4, 1/8, 1/16 or 1/32) (Table 1). Antibody titrations for CD137 (4-1BB) and CD154 were performed on peripheral-blood-mononuclear-cells (PBMCs) that were stimulated with T-activator CD3/CD28 beads (Thermos Fisher Scientific) overnight at 1:10 dilution.

**Table 1:** Antibodies, staining indexes and usage of antibodies in the panels.

Antibody*	Clone	Format	THACT^†^ (μg/ml)	SI-1^‡^	SI-2	SI-3	SI-4	SI-5	AFCPSV^§^ (μg/ml)
CD3	SK7	FITC	6.67	156	158	99.2	69.5	33.6	0.75
CD3	UCHT1	BV510	6.67	347	269	116	59.6	199	0.83
CD3	UCHT1	BUV737	3.33	369	278	196	113	70.6	0.42
CD4	RPA-T4	V450	6.67	183	173	144	95.3	53.4	0.83
CD4	RPA-T4	V500	3.33	76.9	69.3	46.4	28.4	14.4	1.25
CD4	RPA-T4	PE-Cy7	3.33	1285	1126	857	367	200	0.63
CD8	RPA-T8	APC	0.6	548	740	538	363	164	0.05
CD8	RPA-T8	BB515	1.67	1609	1089	446	188	62.4	0.26
CD11c	B-ly6	PE	6.67	167	127	104	78.3	51.9	0.83
CD14	M5E2	PE-Cy7	3.33	55	87.8	58.3	80.2	63.5	0.38
CD16	3G8	V450	13.33	77.9	157	109	72.4	36.6	5
CD16	NKP15	FITC	10	122	134	90.4	55.4	24.6	3.33
CD19	HIB19	BB515	3.33	211	192	130	97.8	45.1	0.75
CD19	HIB19	PE-CF594	3.33	414	524	613	605	529	0.38
CD21	B-ly4	PE	5	304	369	420	457	511	0.38
CD24	ML5	PE-Cy7	3.33	238	178	127	88.3	48.6	0.75
CD25	2A3	BB515	1.67	21	21.5	19.7	14.3	12.2	0.83
CD27	M-T271	PE-CF594	3.33	603	603	375	314	158	0.75
CD27	L128	BV711	6.67	147	166	184	155	132	1.5
CD28	CD28.2	APC	1.67	84.3	72.9	62	42.9	34.6	0.83
CD38	HIT2	BV421	13.32	34.1	32	27.5	21.5	11.8	2.99
CD39	7 TU66	BUV737	6.67	24.4	25.2	19.9	13.7	8.98	3.33
CD45	HI30	BUV395	6.67	59.9	75.6	57.7	53.3	52.1	0.63
CD45RA	L48	PE-Cy7	3.33	106	110	94.3	80	57.8	0.42
CD45RO	UCHL1	BV786	1.67	113	66.3	50.9	31.6	13.2	0.63
CD56	NCAM16.2	PE	1.67	96.1	59.4	144	100.5	78.9	0.31
CD57	NK-1	PE-CF594	6.67	851	742	304	138	67.3	1.25
CD62L	SK11	BV711	13.33	77.1	64.2	42.4	43.6	47.5	1.06
CD64	MD22	APC-R700	1.67	126	157	129	72.4	45.6	0.63
CD88(C5aR)**	D53-1473	PE	13.32	19.9	12.3	4.35	2.61	2.31	14.2
CD123	7G3	PE-CF594	1.67	279	304	263	230	94.7	0.2
CD127	HIL-7R-M21	PE	26.68	83.1	87.8	93.2	94	76.5	2.5
CD127	HIL-7R-M21	PE-CF594	1.67	26.1	19.9	14.6	7.39	3.32	1.25
CD137(4-1BB)**	4B4-1	BV421	6.67	15.6	15.9	13.4	10.5	9.66	0.8
CD141(BDCA3)	A1A4	BV711	1.67	10.4	8.96	9.02	7.56	7.4	0.83
CD154**	TRAP1	APC	0.6	7.55	7.05	6.43	6.11	5.5	0.3
CD183(CXCR3)	1C6	PE-CF594	6.67	37.9	28.3	19.8	12.6	11.2	6.0
CD197(CCR7)*	150503	PE	1.67	20.6	15	11.8	9.77	6.01	1.67
CD303(BDCA2)*	AC144	APC	6.67	96.2	63.4	48.1	27.6	18	3.33
FOXP3	259D/C7	PE-CF594	-	-	-	-	-	-	10
HLA-DR	G46-6	BV510	3.33	177	110	63.9	43.8	27.8	0.83(P3) 1.5(P5)
IgD	IA6-2	BV510	3.33	126	128	122	108	70.4	0.75
IgM**	G20-127	APC	0.6	69.9	50.8	38.9	32.7	25.8	0.4
TCRαβ	T10B9.1A-31	BUV737	3.33	50	29	14.2	6.43	3.63	3.33
TCRγδ	11F2	PE-Cy7	-	-	-	-	-	-	2.0
Lineage (CD3, CD14, CD19,CD20, CD56)	SK7, SJ25C1, L27, MφP9, NCAM16.2	FITC	-	-	-	-	-	-	5.28

*Used antibodies were supplied by BD Biosciences, except CD303 and CCR7 were obtained from Miltenyi Biotec and R&D Systems respectively.

^†^THACT: The highest antibody concentration for titration.

^‡^SI-1: Staining index-1 (SI-1) under the highest antibody concentration. 100μl of anticoagulated-WPB and 160μl total staining volume were used for antibody titration under 5–6 serial dilutions of antibodies (1, 1/2, 1/4, 1/8, 1/16 or 1/32) at room temperature (22°C to 30°C) for 15 minutes. The majority of these antibodies were assessed for their SI on lymphocytes or combined monocytes based on FCS vs SSC. The SI of CD25, CD127, CD154 and 4-1BB on CD3+CD4+ T cells, CD28, CD62L and CCR7 on CD3+ T cells, CD8 on CD4- negative lymphocytes, and CD56 on CD19 negative lymphocytes were assessed.

**The SI of C5aR were assessed on granulocytes. Anticoagulated-WPB was washed with cold PBS twice before IgM staining. The antibody titrations for 4-1BB and CD154 were performed on 1×10^6^ PBMCs that were stimulated with CD3/CD28 beads overnight.

^§^AFCPSV: antibody final concentration in the panel staining volume.

P3: Panel 3

P5: Panel 5

### Multicolour panels on BD-LSR Fortessa

Panel design was based on fluorochrome brightness, antigen density and co-expression, fluorochrome spill over of interested immune-cell subsets and reagent availability in each panel for the 5 laser 18 parameter BD-LSR Fortessa. The finalised absolute cell-count panel and 7 leukocyte panels containing 8–10 antigen markers for monitoring subsets and/or status of granulocytes, monocytes, DCs, NK, B, and T cells including NKT and Tregs, were designed to assess the WPB immune profiling of healthy individuals and monitor islet transplant recipients with T1D (Table 2). Additional tested panels are listed in S2 and S3 Tables.

**Table 2:** The finalised panels on the 5 laser Fortessa.

Laser nm	355		403				488	561			639	
Laser mw	20		50				50	50			40	
Filter	379/28	740/35	440/40	525/50	710/50	780/60	488/10	586/15	610/20	780/60	670/14	730/45
Panel 1 (absolute number count)	CD45 (BUV395)						CD3 (FITC)	CD56 (PE)	CD19 (PE-CF594)	CD14 (PE-Cy7)		
Panel 2 (general immune phenotype)	CD45 (BUV395)		CD16 (V450)	CD4 (V500)			CD3 (FITC)	CD56 (PE)	CD19 (PE-CF594)	CD14 (PE-Cy7)	CD8 (APC)	CD64 (APC-R700)
Panel 3 (DCs)	CD45 (BUV395)		CD16 (V450)	HLA-DR (BV510)	CD141 (BV711)		LIN (FITC)	CD11c (PE)	CD123 (PE-CF594)		CD303 (APC)	
Panel 4 (B cells)	CD45 (BUV395)		CD38 (V421)	IgD (BV510)			CD19 (BB515)	CD21 (PE)	CD27 (PE-CF594)	CD24 (PE-Cy7)	IgM (APC)	
Panel 5 (T cell activation)	CD45 (BUV395)	CD3 (BUV737)	CD4 (V450)	HLA-DR (BV510)	CD27 (BV711)		CD8 (BB515)		CD57 (PE-CF594)	CD45RA (PE-Cy7)	CD28 (APC)	
Panel 6 (naïve and memory)	CD45 (BUV395)		CD4 (V450)	CD3 (BV510)	CD62L (BV711)		CD25 (BB515)	CD197 (PE)	CD127 (PE-CF594)	CD45RA (PE-Cy7)	CD8 (APC)	
Panel 7 (TCRαβ/TCRγδ & T cell state & neutrophils)	CD45 (BUV395)	TCRαβ (BUV737)	CD4 (V450)	CD3 (BV510)		CD45RO (BV786)	CD16 (FITC)	CD88 (PE)	CD183 (PE-CF594)	TCRγδ (PE-Cy7)	CD8 (APC)	
Panel 8 (FOXP3 Tregs)	CD45 (BUV395)	CD39 (BUV737)	CD137 (V421)	CD3 (BV510)		CD45RO (BV786)	CD25 (BB515)	CD127 (PE)	FOXP3 (PE-CF594)	CD4 (PE-Cy7)	CD154 (APC)	

### Standardizing cytometer setup, fluorescence compensation and application settings

Instrument setup and performance tracking was performed daily using BD cytometer setup and tracking (BD CS&T) beads to monitor instrument performance. The instrument specific configuration (laser wavelength, powers, filters) used is listed on Table 2. To create fluorescence compensation settings for panels anti-Mouse Ig, κ/negative control compensation particles (BD Biosciences) were stained with an antibody conjugated to the fluorochrome being compensated and the data used to auto-calculate multiple fluorescence compensations. In panel 6, BV711-CD62L was compensated using WPB sample. In order to ensure consistency of results over time for monitoring samples of pre- and post-transplantation, application settings for each panel were created using BD FACSDiva Version 6 Software (BD Biosciences).

### Direct immunofluorescence staining protocols of anticoagulated-WPB sample

Prior to staining, fluorescent-antibody cocktails for each panel, except the panel used to determine an absolute cell-number, were pre-mixed in BD Horizon Brilliant Stain Buffer (BV buffer) (BD Biosciences) at a 1:2 ratio <60 minutes before staining. Sample staining started within 120 minutes of blood sample collection [2]. Three staining protocols were used.

Staining Protocol 1: For the absolute cell-number panel, 50μl of anticoagulated-WPB was added into a Trucount tube (BD Biosciences), and stained with pure 2.5μl Fc block (Fc1.3070, BD Biosciences) for 10 minutes at room temperature in the dark. 10μl of antibody cocktail was added into the sample and incubated for 15 minutes at room temperature. 450μl 1× BD FACS lysing solution (BD Biosciences) per sample was added and incubated for 15 minutes at room temperature as per the manufacturer’s instructions (BD Bioscience). Samples were then transferred to 4°C for acquisition on a BD-LSR Fortessa (BD Biosciences).

Staining Protocol 2: The second protocol used was for panels 2, 3, 4 (B cell panel), 5 and 6. The B cell panel had an additional step in which 300μl of anticoagulated-WPB was washed with cold 10ml PBS (Astral Scientific) and centrifuged at 300g for 10 minutes twice before Fc block. In this protocol, 100–300μl of anticoagulated-WBP in a FACS tube was blocked with 5–15μl Fc block for 10 minutes at room temperature in the dark, then stained with 100–300μl of antibody cocktails for 15 minutes at room temperature, in the dark. Samples were then lysed and fixed with 1×FACS lysing solution (BD Biosciences) for 10 minutes at room temperature. All following steps were performed at 4°C. The lysed samples were centrifuged and washed twice at 300g for 5 minutes before acquisition to remove excess antibody, lysed RBCs, and platelets.

Staining Protocol 3: The third protocol was for the FOXP3 Treg panel and had the same steps for surface antibody staining as above, using 300μl anticoagulated-WPB. After surface antibody staining, lysis and washing steps, the samples were fixed and permeabilized with transcription factor buffer set (BD Biosciences) for 45 minutes at 4°C, and stained with FOXP3 at 4°C for 40 minutes according to the manufacturer’s instructions. The sample was left to settle on ice for 2 hours before acquisition.

### Data analysis and statistics

Data was acquired using BD FACSDiva Version 6 Software system (BD Biosciences), and was analysed using FlowJo v10. For staining indexes (SI) of titration on each antibody, data was initially presented as median fluorescence intensity (MFI) values for both the positive cell-populations and unlabelled cell-populations (negative). Staining index was calculated using the formula, where SI equal = (positive MFI signal—MFI negative)/[(84^th^ percentile MFI negative–MFI negative)/0.995][25]. Absolute cell-numbers were calculated using the formula, where cells numbers = (number of even in region containing cell / number of even in absolute count bead region) × (number of beads per test/test volume) as per instruction manual (BD Biosciences). Spill over spreading matrix was calculated using FlowJo V10 [26]. A data analysis template for each panel was created using FlowJo V10 and was applied to all individual samples. Coefficient of variation (CV) of selected immune cell counts and population frequencies was calculated using the ratio of standard deviation to mean and multiplied by 100. In order to assess repeatability and reproducibility of the study, two-way and three-way ANOVA were used to partition the variation in immune cell counts and population frequencies and quantify the variance explained by either subject, panel or date of sampling using R v3.5.1.

## Results

### Establishment of 8 leukocyte profiling panels

**Fig 1:**
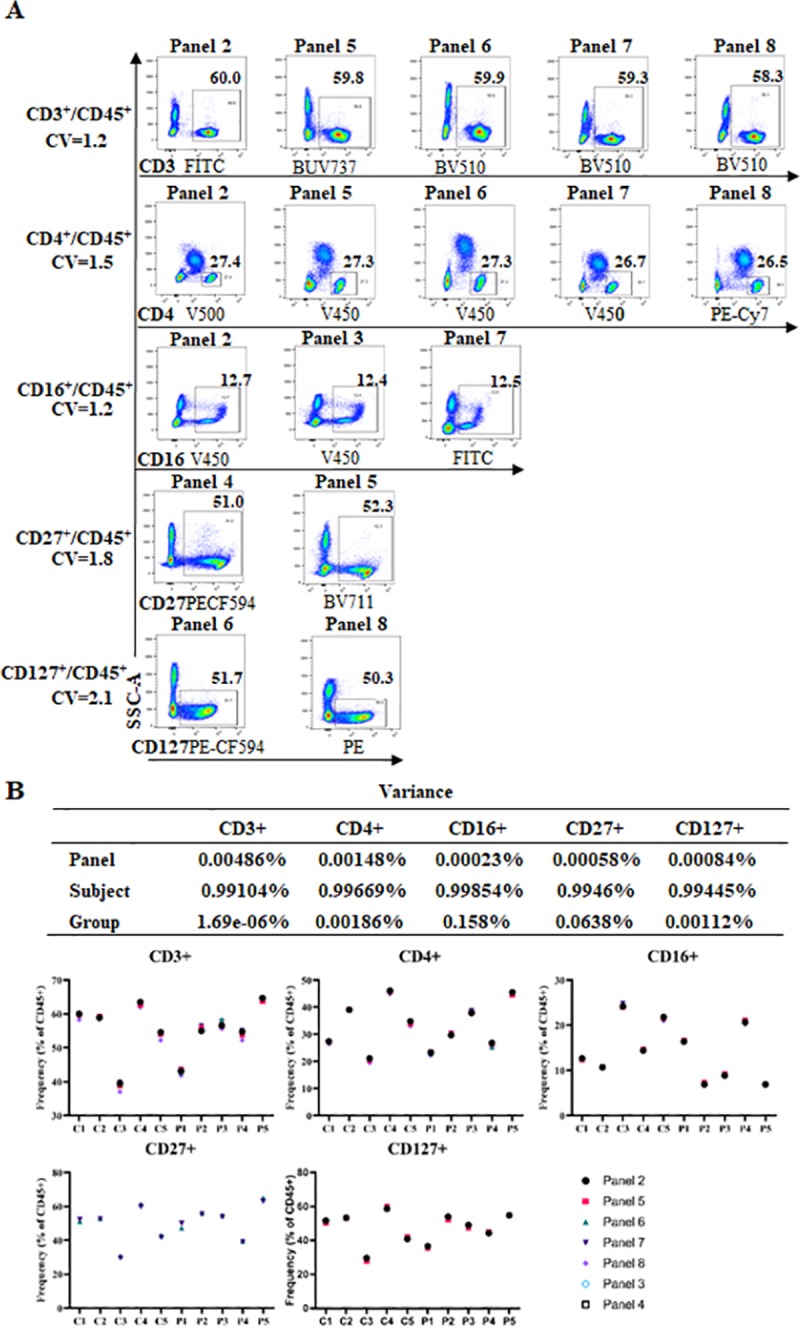
The selected immune cell populations were identifiable across panels. **(A)** CD3 vs SSC-A, CD4 vs SSC-A, CD16 vs SSC-A, CD27 vs SSC-A, and CD127 vs SSC-A plots (after gating on CD45+ but excluding granulocytes) from one healthy control across seven panels (panels 2–8) showed very similar population frequencies for each immune subset. Among these, CD3 with 2 clones (UCHT1, SK7) and three fluorochromes (FITC, V510-CD3, BUV737); CD4 with 3 fluorochromes (V450, V500, PE-Cy7) and one clone (RPA-T4); CD16 with 2 fluorochromes (V450, FITC) and two clones respectively (3G8, NKP15); CD27 with two fluorochromes (PE-CF594, BV711) and two clones respectively (M-T271, L128); CD127 with two fluorochromes (PE and PE-CF594) and one clone (HIL-7R-M21). The CVs of these populations are shown. **(B)** The variation in cell proportions explained by differences across panels (panel 2–8), subjects (n = 10), and groups (control, n = 5 and patient, n = 5) in the proportion frequencies of CD3+ and CD4+ (on panel 2, 5, 6, 7 and 8), CD16+ (on panel 2, 3 and 7), CD27+ (on panel 4 and 5), CD127+ (on panel 6 and 8) in total CD45+ cells for control samples 1–5 and patient samples 1–5 at pre-transplantation.

The absolute cell-count and seven leukocyte panels containing 8–10 antigen markers in each panel with 46 fluorochrome-conjugated antibodies were established on a BD-LSR Fortessa (Tables 1 and 2). Where practical the same antibody was in different panels (such as BUV395-CD45, clone:HI30), whereas in others different fluorochromes and/or different clones were used to minimise spillover spreading to cell populations of interest. For example, CD45-BUV395, which had a SSM of 0–0.277 across all panels and demonstrated minimal spillover spread to other parameters, was used across all panels (S4–S10 Tables). In contrast, CD4 (clone:RPA-T4) used 3 different fluorochrome-conjugated antibodies (V450, V500 and PE-CY7) across 5 panels, while CD8 (APC and BB515, clone:RPA-T8), CD19 (BB515 and PE-CF594, clone:HIB19), and CD127 (PE and PE-CF594, clone:HIL-7R-M21) each had 2 types of fluorochrome-conjugated antibodies with the same clone (Tables 1 and 2). CD3 was detected with 3 different fluorochromes and 2 different clones (BV510 and BUV737, clone:UCHT1; FITC, clone:SK7) across 6 panels (Tables 1 and 2). Importantly, individuals had similar population frequencies (for CD3+, CD4+, CD16+, CD27+ and CD127+ in CD45+ cells (excluding granulocytes) when measured using different panels for individual subjects both in the healthy control (C, n = 5) and pre transplantation patient (P, n = 5) (S11 Table and Fig 1). The CVs of these populations are listed in S11 Table. The variation of these frequencies across panels for CD3+, CD4+, CD16+, CD27+ and CD127 was 0.00486%, 0.00148%, 0.00023%, 0.00058%, and 0.00084% respectively (Fig 1B). Moreover, the majority of the variation in cell frequencies could be explained by the differences between individuals explaining 0.99104%, 0.99669%, 0.99854%, 0.9946% and 0.99445% of the variation in CD3+, CD4+, CD16+, CD27+ and CD127 cell frequencies respectively (Fig 1B). This data demonstrated across-panel comparisons were possible and identifiable although the antibodies used across the panels were not identical.

The technical repeatability was performed on three controls in panel 1 with five repeated-tests for each control sample. The CVs of absolute cell numbers of granulocytes, monocytes, B cells, T cells, NK cells, and NKT cells in three controls were ≤4.3, ≤4.7, ≤3.9, ≤2.7, ≤3.6 and ≤4.9 respectively (S12 Table and S1 Fig). The variations of these cell numbers across five repeated-tests on each control sample for granulocytes, monocytes, B cells, T cells, NK cells, and NKT cells were relatively small, 0.000943%, 0.003851%, 0.00252%, 0.000758%, 0.000577% and 0.000596% respectively (S1B Fig). Moreover, the majority of the variation could be explained by the differences between individuals, with the differences between individuals explaining 0.973832%, 0.967796%, 0.989188%, 0.988883%, 0.998214% and 0.99657% of the variation in granulocytes, monocytes, B cell, T cell, NK cell, and NKT cell numbers respectively (S1 Fig). Together, the results showed technical repeatability in this study.

### The best staining indexes of 43 antibodies on WPB

**Fig 2:**
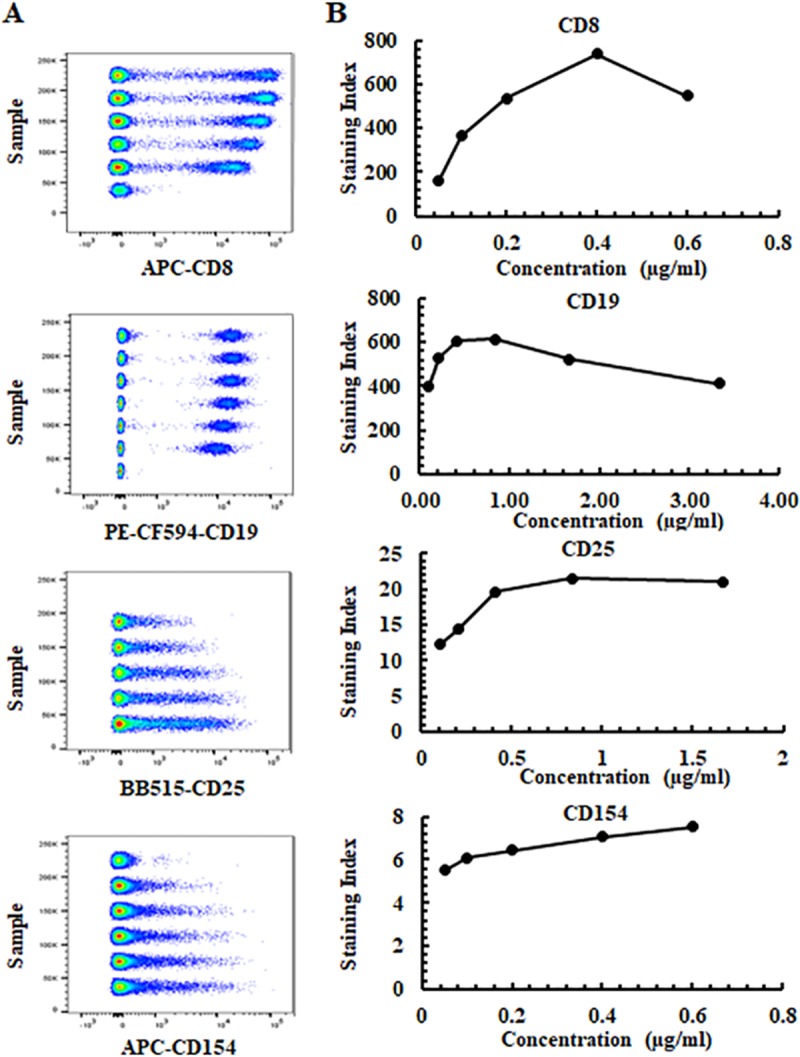
Four representative fluorochrome-conjugated antibody titrations. **(A)** Concatenated pseudocolor data at 5–6 antibody concentrations for APC-CD8 (RPA-T8), PE-CF594-CD19 (HIB19), BB515-CD25 (2A3) and APC-CD154 (TRAP1)**. (B)** Staining indexes and antibody concentrations for APC-CD8, PE-CF594-CD19, BB515-CD25 and APC-CD154. APC-CD8, PE-CF594-CD19, BB515-CD25 were assessed on lymphocytes (excluding CD4+ cells), lymphocytes or CD3+CD4+ T cells respectively. APC-CD154 was evaluated on CD3+CD4+ T cells of PBMCs that were activated with CD3/CD28 beads overnight. The final antibody concentrations used in the study for APC-CD8, PE-CF594-CD19, BB515-CD25 and APC-154 were 0.05 μg/ml, 0.38 μg/ml, 0.83 μg/ml, 0.3 μg/ml respectively.

The positive and negative MFI signals for the majority of antibodies were evaluated on lymphocytes or combined with monocytes based on FCS vs SSC for calculation of the staining index. These SI values were calculated as indicated in the methods section and are listed in Table 1. To obtain the SI of CD25, 5 serial dilutions of BB515-CD25 combined with the same concentration of BV510-CD3, V450-CD4 and PE-CF594-CD127 were used, then a MFI of BB515-CD25 positive and negative cells were assessed based on CD3+CD4+ cells (Fig 2 and Table 1). The best SI of BB515-CD25 was 21.5 using the 0.83 μg/ml antibody concentration (Table 1 and Fig 2). The MFI of PE-CD127 was evaluated on CD3+CD4+ T cells, and the SI of PE-CD127 were 93.3 and 94 at a antibody concentration of 6.7μg/ml (SI-3) and 3.3μg/ml (SI-4) respectively (Table 1). The best SI of PE-CF594-CD127 evaluated on CD4+ lymphocytes was 26.1 using an antibody concentration of 1.67μg/ml (Table 1). The positive and negative MFI of CD28, CD62L and CCR7 were based on their expression on CD3+ T cells, while CD8 was based on expression targeting lymphocytes (excluding CD4+ cells), and CD56 expression based on lymphocytes (excluding CD19+ cells) (Fig 2 and Table 1). The positive and negative MFI of BV421-CD137 and APC-CD154 were evaluated on CD3+CD4+ T cells of PBMCs that were activated with CD3/CD28 beads overnight. The best SI of BV421-CD137 was 15.9 using an antibody concentration of 3.3μg/ml and the best SI of APC-CD154 was 7.55 with an antibody concentrations of 0.6μg/ml (Fig 2 and Table 1). It is important to note that the staining indexes calculated are specific to the instrument configuration. Additionally, not all antibodies reached saturation using the concentrations tested (Fig 2 and Table 1). Antibodies that are not used at saturating concentrations may show differences due to differing cell-numbers present between samples.

### Establishment of antibody quantity in panel-cocktail

**Fig 3:**
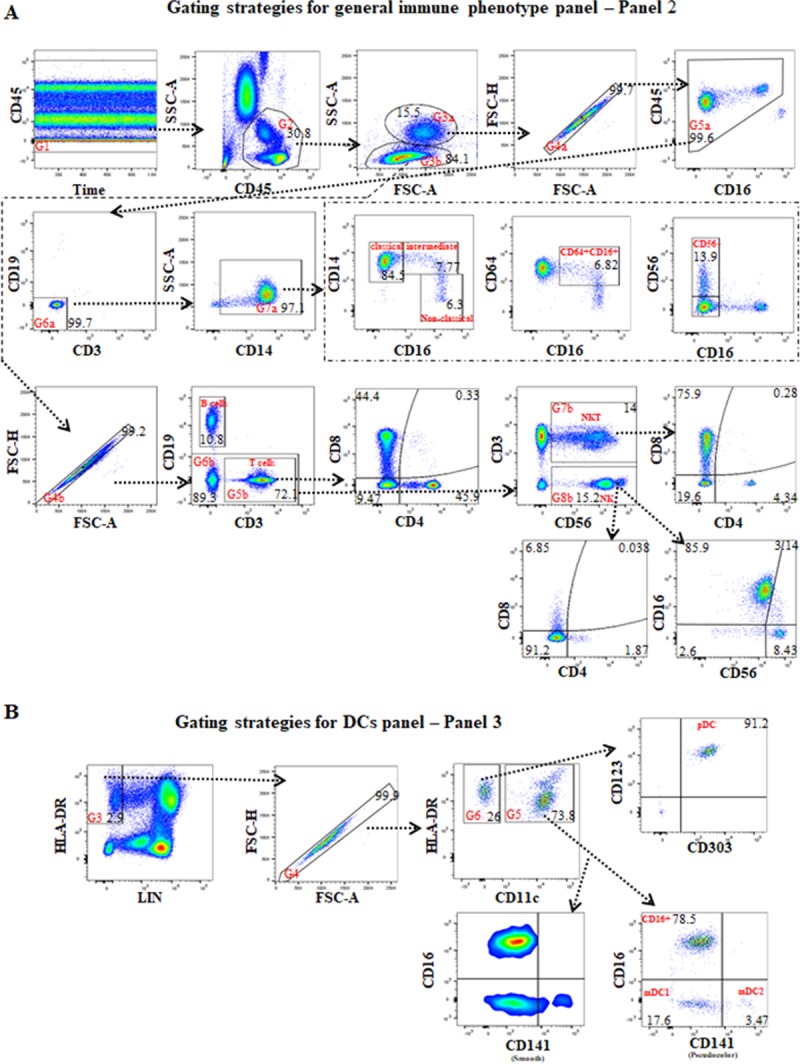
The gating strategies for the general immune phenotype panel (panel 2) and the DC panel (panel 3). **(A)** For panel 2, the first gate (G1) is on time vs CD45 to exclude poor flow, the second gate (G2) is on CD45 vs SSC-A to exclude granulocytes, the third gate (G3) is on FSC-A vs SSC-A for separating monocytes, including DCs (G3a), and lymphocytes (G3b). Under G3a is G4a which is on FCS-A vs FSC-H to exclude double-cells, G5a is on CD16 vs CD45 to exclude granulocytes again, G6a is on CD3 vs CD19 to exclude T and B cells, G7a is on CD14 vs SSC-A for CD14+monocytes, with further assessment of classical (CD14+brightCD16-), intermediate (CD14+brightCD16+), non-classical (CD14+dimCD16+bright); CD16+CD64+(CD16 vs CD64); and CD56+CD16- (CD16 vs CD56) monocytes. Under G3b is G4b which is on FCS-A vs FSC-H to exclude double-cells on lymphocyte. CD3 vs CD19 under G4b is for B and T cells populations. Gating on CD3+ T cells (G5b) was done to further assess CD4+, CD8+, CD4-CD8- and CD4+CD8+ subsets (CD4 vs CD8); gating on CD19- cells (G6b) was undertaken to assess CD3+CD56+ NKT cells and CD56+ NK cells (CD56 vs CD3); gating on CD3+CD56+ NKT (G7b) was done for assessment of CD4 vs CD8 subsets of NKT cells. Gating on CD56+ NK in gate G8b further assessment of CD16+CD56+, CD16+CD56+bright (intermediate), CD16-CD56+bright and CD16-CD56+ NK subsets (CD56 vs CD16), and CD4+, CD8+, and CD4-CD8- NK subsets (CD4 vs CD8) was undertaken. **(B)** The gating strategies for the DC panel (panel 3). The first and second gates were the same as in panel 2. Gating on HLA-DR+LIN- (CD3CD14CD19CD20CD56) in gate G3 (LIN vs HLA-DR) was performed to exclude CD14+ monocytes, CD3+ T, CD19+ and CD20+ B, CD56+ NK cells, then the fourth gate (G4) was performed to exclude double-cells. Gating on CD11c+HLA-DR+ cells in gate G5 was performed to assess CD141+mid/bright mDC2, CD141- mDC1 and CD16+ DCs (pseudocolor and smooth of CD141 vs CD16); and gating on CD11c-HLA-DR+ cells (G6) was undertaken to assess plasmacytoid DCs CD303+CD123+ (pDCs) and CD303-CD123-/dim HLA-DR+ immature cells (CD303 vs CD123).

**Fig 4:**
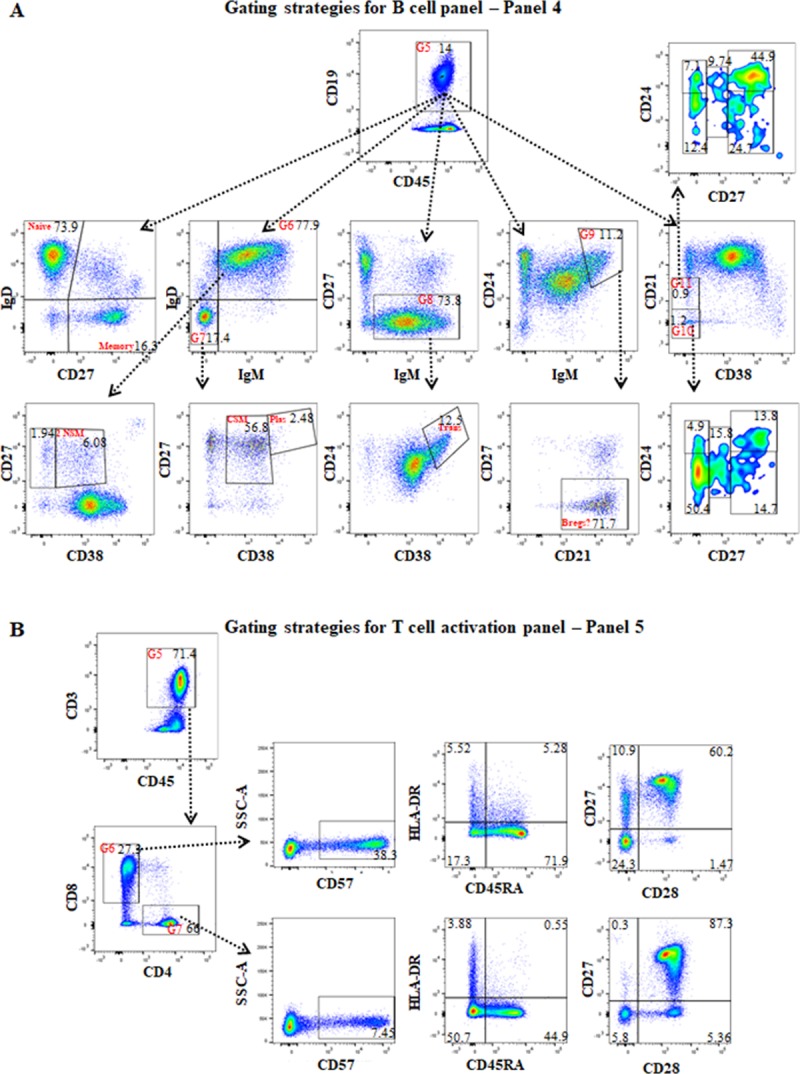
The gating strategies for B cell panel (panel 4) and T cell activation panel (panel 5). **(A)** For panel 4, the first and second gates were the same as panel 2 (Fig 3), the third gate was on lymphocytes based on FSC-A vs SSC-A, and the fourth gate was on FCS-A vs FSC-H to exclude double-cells as was done in panel 2. After gating on CD19+ B cells (G5), using CD27 and IgD expression identified IgD+CD27- naïve and CD27+IgD- memory B cells; after gating on IgM+IgD+ (G6), using CD38 and CD27 expression identified CD27+CD38+ and CD27+CD38- non-switched memory B cells; after gating on IgM-IgD- (G7), using CD38 and CD27 expression identified CD27+CD38+bright plasmablasts (Plas) and CD27+CD38+dim class switched memory (CSM) B cells; after gating on IgM+CD27- B cells, visualising CD38 vs CD24 identified CD24+brightCD38+bright transitional B cells (Trans); after gating on IgM+brightCD24+bright, using CD21 and CD27 expression identified CD21+CD27- B cells that may be regulatory B cells; gating on CD38-CD21- (G10) and CD38-CD21low (G11) identified innate-like or memory B cells (CD38 vs CD21) with gating using CD27 and CD24 expression for assessment of CD24+brightCD27-, CD24low/-CD27, CD27low, CD27+brightCD24+bright, and CD27+brightCD24low/- B cells. **(B)** For panel 5, the first and second gates were the same as in panel 2, and the third and fourth gates were the same as panel 4. After gating on CD45+CD3+ T cells (G5), CD4 vs CD8 identified the T cell subsets. Gating on CD8 T cells (G6) or CD4+ T cells (G7), comparisons of CD57 vs SS-A, CD45RA vs HLA-DR, and CD28 vs CD27 were performed for assessment of T cell activation.

**Fig 5:**
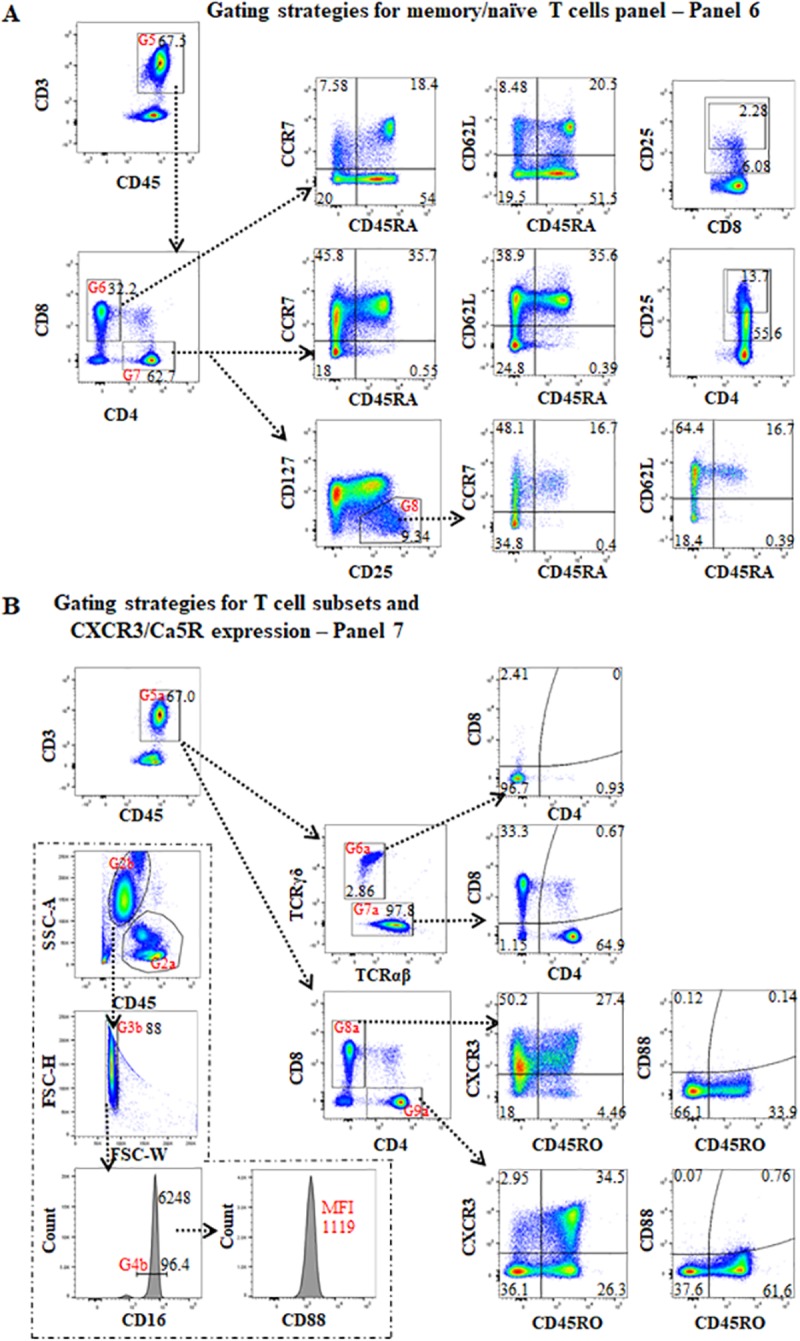
The gating strategies for memory and naïve T cell panels (panel 6), T cell subsets, and CXCR3 and Ca5R expression (panel 7). **(A)** For panel 6, the strategies of the first to fourth gates were the same as panel 4 (Fig 4). After gating on CD3+CD45+ T cells (G5) and then gating on CD8 T cells (G6) or CD4+ T cells (G7), CD45RA vs CCR7 and CD45RA vs CD62L identified naïve (CD45RA+CCR7+/CD62L+), central memory (CD45RA-CC7+/CD62L+), effector memory (CD45RA-CC7-/CD62L-) and terminal differentiated effector memory (TEMRA) (CD45RA+CC7-/CD62L-) CD4+ or CD8+ T cells. CD8 vs CD25 and CD4 vs CD25 can be used to estimate the ratio of CD8+CD25+, CD8+CD25+bright, CD4+CD25+ and CD4+CD25+bright T cells respectively. To identify Treg cells, the initial gate is on CD4+ T cells (G7), then evaluation of CD25 vs CD127 expression with the CD25+CD127-/dim population defined as Treg cells. Further gating on these Tregs (G8), and gating on CD45RA vs CCR7 and CD45RA vs CD62L separated CD45RA+CCR7+/CD62L+ naïve-Tregs from CD45RA-CCR7+/CD62L+ and CD45RA-CCR7-/CD62L- activated Tregs. **(B)** For panel 7, the first part focuses on CD3+ T cells. The first to fourth gates (G1-G4) were the same as in panel 6. After gating on CD3+CD45+ T cells (G5a), TCRαβ vs TCRγδ showed the proportion of TCRαβ and TCRγδ in CD3+ T cells. Gating on TCRγδ (G6a) and TCRαβ (G7a), CD4 vs CD8 showed the proportion of CD4+, CD8+, CD4-CD8- and/or CD4+CD8+ T cells on these two TCR cell-populations. Additionally, after gating on CD3+CD45+ T cells (G5a), then gating on CD8+ (G8a) or CD4+ (G9a) T cells on CD4 vs CD8, expressions of CXCR3 and C5aR on CD45RO+ and CD45RO- of CD8+ and CD4+ T cells were assessed on CD45RO vs CXCR3 or CD45RO vs C5aR respectively. In the second portion of the panel, the first gate was the same as panel 2 (Fig 3). After gating for granulocytes (G2b) (CD45+dimSSC-Ahigh), the third gate (G3b) was on FCS-W vs FSC-H to exclude double-cells, the percentage of CD16+bright cells on the histogram of CD16 represents neutrophils, and the MFI of CD16+bright cells was measured. After gating on neutrophils (G4b), the histogram of CD88 (C5aR) expression was assessed by MFI.

**Fig 6:**
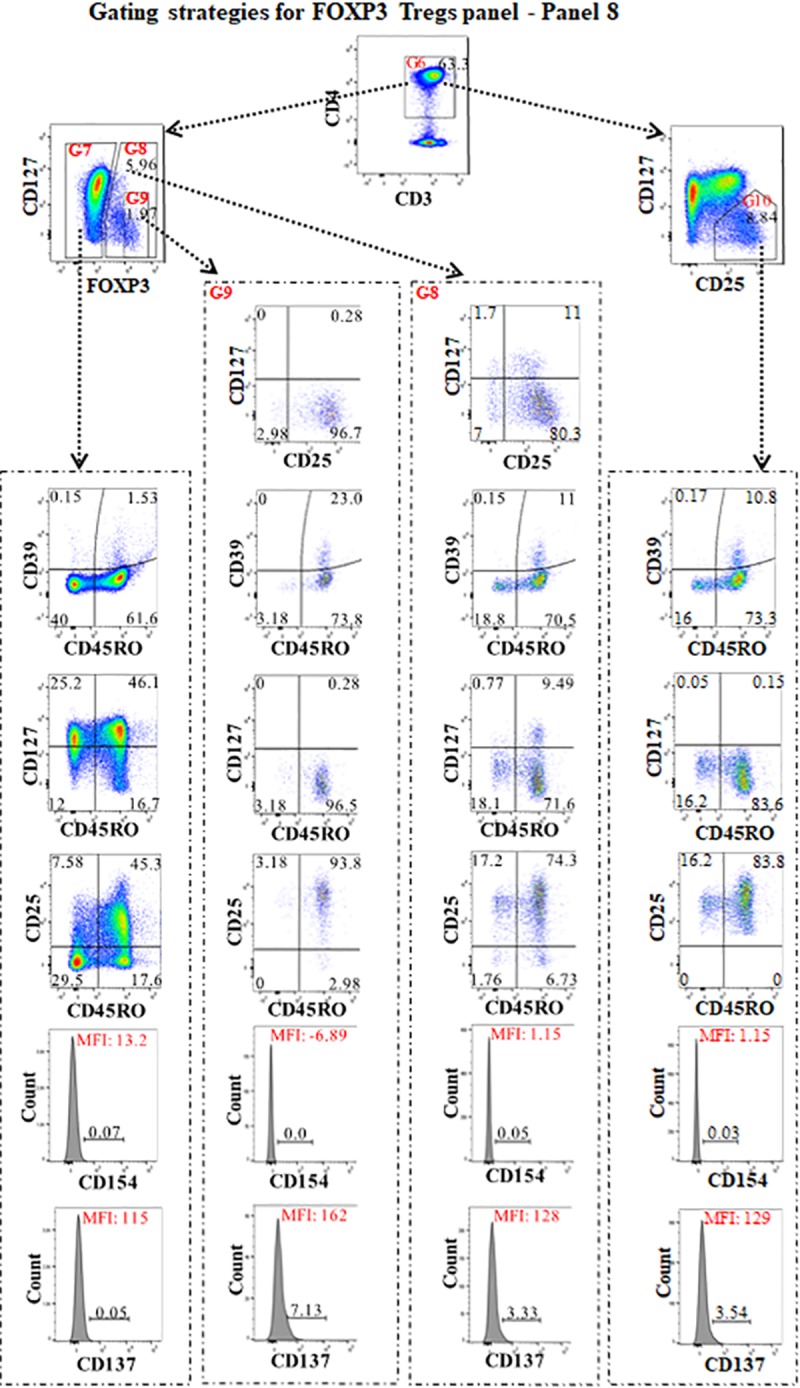
The gating strategies for identifying FOXP3+ Tregs (panel 8). For panel 8, the strategies of the first to fifth gates were the same as in panels 5 and 6 (Figs 4B and 5A). After gating on CD3+CD4+ T cells (G6), FOXP3 vs CD127 was used to assess the proportions of CD4+FOXP3- effector cells (G7), CD4+FOXP3+Tregs (G8) and CD4+FOXP3+bright (G9) Tregs. CD25 vs CD127 were used to assess CD25+CD127-/dim Tregs. After gating on CD4+FOXP3- effector cells (G7), CD4+FOXP3+ Tregs (G8), CD4+FOXP3+bright Tregs (G9), and CD25+CD127-/dim (G10), CD45RO vs CD39, CD45RO vs CD127, and CD45RO vs CD25 were used for assessment of CD39, CD127 and CD25 expression on memory (CD45RO+) or naïve (CD45RO-) non-FOXP3 effector T cells, and CD4+FOXP3+, CD4+FOXP3+bright, or CD25+CD127-/dim Tregs. Gating on FOXP3+ (G8) and FOXP3+bright (G9) Tregs, and gating on CD25 and CD127 identified CD25+CD127-/dim, CD25+CD127+, CD25-CD127- and CD25-CD127+ on Foxp3+/brightTregs. The histogram of CD137 and CD154 was assessed on these cell types.

Antibody usage for each panel was based on SI (Table 1), fluorochrome brightness, antigen density and co-expression, SSM (S4–S10 Tables) and gating strategies for immune-cell subsets (Figs 3–6). The final concentration of each antibody for the total staining volume in the panels are listed in Table 1. For example, the SSM of APC-R700-CD64 to PE-Cy7-CD14 in panels 2 was 3.01 (S4 Table). In this panel, we gated on CD14, then assessed the proportion of CD16+CD64+ monocytes (Fig 3). In order to reduce the spillover effect, the final concentration of 0.63μg/ml was used rather than the concentration that produced the best SI (0.83μg/ml), as this was an easily identifiable population where the MFI was not analysed (Table 1). The SSM of PE-CF594-CD19 to PE-CD56 was 1.21 (S4 Table). This spillover spreading had minimal effect due to CD19+B cells and CD56+NK cells being separated on lymphocytes. Considering the expression of CD19 separated clearly on lymphocytes, the final concentration of 0.38μg/ml for the PE-CF594-CD19 antibody was used, which was less than the concentration that produced the best SI (0.42μg/ml), in order to minimize spill over from this fluorochrome and minimize antibody usage (Table 1). Since CD25 has a range of expression on CD4+ T cells with CD25+dim, mid to bright cell-populations, and the SSM of BB515-CD25 for each parameter in panel 6 and panel 8 was <0.414 (sum: 0.461–0.503) (S8 and S10 Tables), the final concentration of 0.83μg/ml for the BB515-CD25 antibody was used in the panels (Table 1). This was shown to produce the best SI (SI-2, 21.5) (Fig 2). CD154 on T cells is expressed after activation (Fig 2), and the SSM of APC-154 to PE-cy7-CD4 and PE-CF594-FOXP3 was 0.854 and 0.121 respectively (S10 Table). As a result, a final concentration of 0.3μg/ml of APC-CD154 (SI-2, 7.05) was used in the panel 8, and not 0.6μg/ml (SI-1, 7.55) to minimize spillover effects on CD4 and FOXP3 cell populations (Table 1).

### Setting analysis templates and gating strategies for target subsets of immune-cells

The analysis templates for gating and the statistic tables were created for each panel in FlowJo. Gating strategies for calculating the absolute cell count panel for each of the major cell subsets (panel 1) are presented in S1A Fig. Absolute numbers of granulocytes, CD3+T cells, NKT cells, 19+B cells, CD14+monocyte, CD56+NK, NKT were measured using this panel.

As well as calculating the absolute numbers for each of the major cell populations the gating strategies enable extensive subclassification of each of these cell types. After gating to identify the general immune phenotype (panel 2) (Fig 3A) an extensive assessment of the proportions and/or subsets of CD14+monocytes, T cells including NKT cells, B cells, and NK cells was undertaken. The subsets of CD14+monocytes contain classical, intermediate, non-classical, CD16+CD64+, and CD56+ monocytes [27]. In addition, the subsets of NK cells including CD16+CD56+, CD16+CD56+bright (intermediate) [28], CD16-CD56+bright and CD16-CD56+dim, and CD4+NK and CD8+ NK[29, 30] were evaluated.

Although DC make up a relatively small proportion of peripheral blood leukocytes they can be identified as shown in panel 3 of Fig 3B. CD16+DCs, myeloid DCs type 1 (mDC1), mDC2 [31, 32], plasmacytoid DCs (pDC)[33], and CD303-CD123-/dimHLA-DR+ immature cells [34] could be accurately measured from this panel.

B cells make up 3–10% of peripheral blood leukocytes and can be divided into naïve, memory, plasmablast and transitional B cells. Gating strategies for the CD19+B cell and B cell subtypes is presented in Fig 4A [35–37]. These included IgD+CD27-naïve, CD27+IgD-memory, two types of non-switched memory (IgD+IgM+CD27+CD38+/CD38), plasmablasts (IgD-IgM-CD27+CD38+bright), class switched memory (IgD-IgM-CD27+CD38+dim), and transitional B cells (IgM+CD27- CD24+CD38+bright). A subset of IgM+brightCD24+bright B cells that were CD21+CD27-B cells and considered Bregs was identified (Fig 4A). The sensitivity of this assay was highlighted by the example shown in Fig 4A (row 2, column 5). The small population of CD38-CD21+dim and CD38-CD21- B cells, described as a subset termed innate-like [38] or memory B cells [39], could be further subdivided into 5 sub-populations based on expression of CD24 and/or CD27.

T cells are the major cell type involved in the initiation and activation of acute rejection after transplantation and current immunosuppressive strategies are focused on either deleting of suppressing the T cell responses. Hence being able to measure the number (Panel 1) and activation status of T cell subsets would be an important tool for evaluating new immunosuppressive strategies. The strategies for evaluating the status of activated T cells is presented in panel 5 (Fig 4B). The expression of CD57 and HLA-DR, and loss of CD27/CD28 expression on both CD4+ and CD8+ T cells were assessed as markers of T cell activation [2, 40–44].

Gating strategies for the evaluation of naïve and memory T cells (panel 6) are shown in Fig 5A. After gating on CD3+CD45+ cells, naïve (CD45RA+CCR7+/CD62L+), central memory (CD45RA-CCR7+/CD62L+), effector memory (CD45RA-CCR7-/CD62L-) and terminal differentiated effector memory (TEMRA)(CD45RA+CCR7-/CD62L-) were evaluated on both CD4+ and CD8+ T cells by the expression or loss of CD45RA combined with the expression or loss of CCR7 and CD62L [45]. Meanwhile, the ratio of CD4+CD25+ with CD4+CD25+bright, and CD8+CD25+ with CD8+CD25+bright, were evaluated. Furthermore, CD25+CD127- Tregs, and CD45RA+CCR7+ naïve-Tregs, CD45RA-CCR7+/CD62L+, and CD45RA-CCR7-/CD62L- memory-Tregs (row 3, column 2 and 3) were assessed [46–49].

In panel 7, the proportion of TCRαβ and TCR γδ in CD3+ T cells and expression of CD4 and CD8 on these two types of cells were assessed (Fig 5B). The expression of CXCR3 and CD88 (C5a anaphylatoxin receptor) (C5aR) were assessed on naïve (CD45RO-) and memory (CD45RO+) CD4+ and CD8+ T cells in order to evaluate T cell activation status after exposure to allo-antigens [50–52]. Since myeloid blood cells have high CD88 expression and unstimulated T cells rarely express CD88, CD88 MFI on neutrophils (CD16+bright) was used to assess the state of neutrophil function [53, 54] and as an internal control for CD88 expression on T cells (Fig 5B).

Gating strategies for identifying FOXP3+ Treg (panel 8) are shown in Fig 6. In this panel, the proportion of CD4+Foxp3+Tregs was first compared to CD4+CD25+CD127dim/-Tregs [55]. The expression of CD25, CD39, CD127, was then assessed on CD45RO+memory and CD45RO-naïve Tregs [56], and effector CD4+ (FOXP3-) as well as FOXP3+bright Tregs. In addition, CD154 and CD137 were assessed on both the Tregs and effector CD4+ (FOXP3-) subsets [57].

### Analysis of immune subsets over time was consistent and reproducible in islet transplant patients

**Fig 7:**
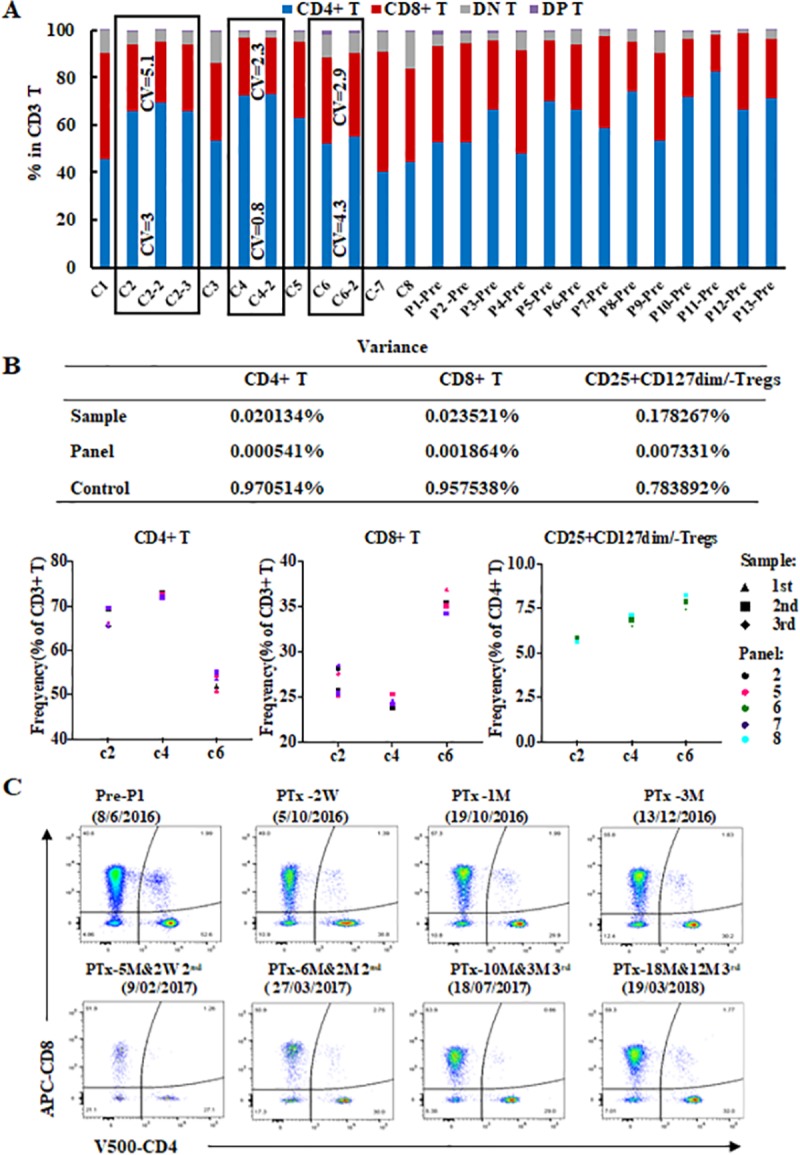
Evaluation of T subsets were consistent and reproducible over a 21 month period. **(A)** The percentage of CD4+, CD8+, CD4+CD8+, CD4-CD8- within the total CD3 T cell population in 12 samples of 8 healthy controls and 13 T1D patients pre-islet transplantation (panel 2). Three healthy-controls (C2, C3 and C6—bold rectangles) had data at 3 or 2 separate time points more than 3 months apart. The CV values of CD4+ and CD8+ T cell frequencies were 3 and 5.1 for C2, 0.8 and 2.3 for C3, and 4.3 and 2.9 for C6 respectively among the different time points (≥2 time points). **(B)** The variances in the proportion frequencies in CD4+ and CD8+ T cell, and CD25+CD127- Treg population frequencies explained by differences on control sample over time (2 or 3 time points) and across panels (panel 2, 5 and 7 for CD4+ and CD8+ T cells, and panel 6 and 8 for CD25+CD127dim/- Tregs), and between controls (C2, C4 and C6) using three-way ANOVA. **(C)** The comparison of CD4 vs CD8 in panel 2 from patient 1 (P1) pre-transplantation, 2 weeks (W), 1 and 3 months (M) after the first islet transplantation, and 2 weeks, 2 months after the second islet transplantation, and 3 and 12 months after the third islet transplantation which occurred across a 21 month time period. This showed that the four subsets of CD3+ T cells (CD4+CD8-, CD8+CD4-, CD4+CD8+, CD4-CD8-) were consistently detected pre and post-transplantation.

In order to determine whether the assessment of immune subsets was consistent and reproducible over time, repeated immunophenotyping of 8 healthy controls as well as 4 recipients on the islet transplant waiting list was undertaken over a 26-month period (Figs 1 and 7, S2–S4 Figs). Three of the healthy-controls (C2, 4 and 6) had repeated samples taken at separate time points, more than 3 months apart. In panel 2, the similar frequencies of CD4+ and CD8+ T cells in CD3+ T cells across different time points (≥ 2 time points) on individual samples were observed in the three controls, and the CVs of CD4+ and CD8+ T cell populations that were measured in the three controls across different time points, were ≤ 4.3 and ≤ 5.1 respectively (Fig 7A). The CVs of CD4+ and CD8+ T cell populations across different time points in panel 5 and 7 also on the three controls were similar as in panel 2 (S13 Table). In panel 6 and panel 8, the CV values of CD25+CD127dim/- Tregs cell frequencies, a low-frequency cell population, were similar to the CD4+ and CD8+ T cell populations (S13 Table). The variance of these frequencies on samples at different time points for CD4+ T cells, CD8 T cells, and CD25+CD127dim/- Tregs was <0.1%, (0.020134%, 0.023521% and 0.178267% respectively). Moreover, the explained variance of CD4+ T cells, CD8+ T cells, and CD25+CD127dim/- Tregs across panels was <0.1%, (0.000541%, 0.001864% and 0.007331% respectively). Furthermore, the variation in cell frequencies between individuals was 0.970514%, 0.957538% and 0.783892% of the variation in CD4+ T cells, CD8+ T cells and CD25+CD127- Treg frequencies respectively (Fig 7B). Finally, the CVs of low-frequency cell populations including HLA-DR+CD45RA- on CD4+ T cells in panel 5, TCR γδ CD3+ T cells in panel 7, CD127+CD45RO+FOXP3+ Tregs in panel 8, CD56+brightCD16- NK cells and CD56+CD16-CD14+ monocytes in panel 2, mDC2 on CD11c+HLA-R+DCs in panel 3 and CD27+IgD- memory on CD45+CD19+ B cells in panel 4, were measured in the three controls across different time points with percentages of ≤6.6, ≤14.3, ≤11.6, ≤14.3, ≤11.3, ≤11.3 and ≤5.5 respectively (S14 Table). Together, this data indicated that evaluation of subject phenotypes were reproducible using this method.

Whilst the CD4+/CD8+ ratios varied from person to person, seven of the eight controls and the patients at pre-transplantation time points showed all showed a CD4+/CD8+ T cell ratio greater than 1, (Fig 7A). The CD4+, CD8+, CD4+CD8+, and CD4-CD8- T cell subsets pre transplant and up to 18 months post-transplant (across 26-months period) in the 4 islet transplant recipients (Patient 1, 2, 4 and 10) were detected consistently (Figs 7C and S2 and S3). After anti-thymocyte globulin (ATG) induction therapy there was a reduction in the numbers of all T cell subsets, consistent with the known T cell depleting effect of this therapy (S2 Fig). In addition there was a reversal of the CD4/CD8 T cell ratio post-transplantation (except patient 1 at 1 month 3^rd^ post transplants and patient 10 at 6 months post transplantation), in all 4 islet transplant recipients evaluated (S2 and S3 Figs). By way of another example, data from the B cell panel (panel 4) of an islet recipient (patient 2) showed that IgD+CD27- naïve and CD27+IgD- memory CD19+ B cells were consistently detected, whilst a change in proportion was identified pre and post-transplantation across a 26 month period (S4 Fig).

### Additional tested antibodies and multicolor panels

Additional antibodies for multiple clones or multiple fluorochromes were tested before finalizing our panels to ensure the best panel was used (S1 Table, and additional tested multicolor panels are listed in S2 Table). For example, among the three clones of the PE-CD56 antibody tested, clone NCAM16.2 showed the best separation of CD56+bright and CD56+dim proportions when compared to clone My31 and B519 in panel 2, where all three antibodies were used at their best SI concentration (S5A Fig). In panel 3, antibody cocktails containing BV711-CD141 (1A4) and APC-CD141 (AD5-14H12) antibody (Miltenyi Biotec) demonstrated that the clone 1A4 of CD141 antibody was nearly identical to the clone AD5-14H12 of CD141 antibody for identification of mDC2 which had CD141+mid and CD141+bright cell populations. Meanwhile, both APC-conjugated and BV711-conjugated CD141 antibodies in the tested panel 3 had better separation than FITC-conjugated CD141 (AD5-14H12) for identification of mDC2 (S6 Fig and S2 Table). It is also worth noting that the fixation/permeabilization procedure with transcription factor buffer reduced BV650-CD127 expression on CD4+ T cells compared to not performing the fixation/permeabilization procedure and this impacted identification of CD25+CD127- Tregs (S5B Fig).

## Discussion

In the current study, we established a method to calculate the absolute immune cell-count and to monitor subsets and states of immune cell activation using eight leukocyte-profiling panels by flow cytometry analysis on a BD-LSR Fortessa. A major advantage of the procedure was that it used less than 1.5 ml anticoagulated-WPB for monitoring the WPB immune-profile in healthy-controls and patients with T1D pre- and post-islet transplantation. We standardized sample handling, reagents, antibody cocktails, staining protocols, instrument setup and data analysis. With this protocol, it was possible to evaluate extensive leukocyte subsets simultaneously and to monitor them longitudinally in clinical trial patients over a period of 26 months.

The advantage of using the BD-LSR Fortessa is that it is possible to design panels with up to 16 colours. In designing our panel we have chosen to limit the number of colours to 8 for several reasons. By reducing the number of colours and increasing the number of detectors provides increased choice for selecting fluorochromes with minimal detector spillover resulting in improved population resolution and decreased inaccuracy. This provides for easier automated gating strategies and bulk analysis workflows. As panel parameter increases, the ability to delve deeper into the data and phenotype of increasingly complex sub-populations also increases. Ideally, more events are required in order to achieve statistical robustness in such small total event population frequencies. With a 10 colour panel, in transplant patients, we were required to increase starting WPB volume from 100μl to 200 μl (Dc panel and Panel 7) or 300 μl (FOXP3 Tregs panels) in order to continue to identify populations. Despite using a high parameter 8 laser cytometer we were limited by gating depth & WPB volume and we prioritised targeted ‘lite’ panels. Finally, a 10 color panel may be more practically compatible between centres and more readily adopted for clinical use as many clinical laboratories are not using high parameter flow cytometry instruments.

The Human Immune Phenotyping Consortium (HIPC) successfully developed five immunophenotyping panels consisting of 8 fluorochrome antibody cocktails to assess immune phenotype of T cells, Treg,Th1/2/17, B cells, and NK/dendritic cells/monocytes on PBMCs in clinical studies [58]. In addition to the HIPC developed panels, panel 8 in this study utilised intranuclear staining to identify FOXP3, a key transcription factor for thymic derived Tregs. Compared to traditional staining methods on PBMCs, where the isolation procedure results in loss of granulocytes, plasmablasts, DCs and monocytes, and requires more blood and more time for sample preparation, the method outlined in the current study requires minimal volume of blood and captures accurate immune-cell information. Previously, the ‘ONE Study’ consortium used WPB for 6 leukocyte-profiling panels, containing 7–9 fluorochrome parameters each, on the 3 laser Navios (Beckman Coulter) for monitoring these immune-cell subsets [2]. Conversely, in our study, we required less than 1.5 ml WPB and obtained accurate immune-cell profiles with 8 panels, containing 8–10 antigen markers in each panel, totalling 46 fluorochrome-conjugated antibodies, and using 12 parameters across 5 lasers on a 18 parameter, 5 laser instrument for monitoring absolute cell-counts, and subsets or status of granulocytes, monocytes, dendritic cells (DCs), B, NK, and T cells including NKT and FOXP3+Tregs.

Antibody titration is crucial to optimize resolution and to obtain robust results for population identification and expression level measurements for multicolour panels. Titration allows for calculation of SI values and to optimize the concentration of antibody for the best separation of leukocyte sub-populations in WPB samples and, where possible, to use less antibody than recommended by the supplier [25] while identifying populations where sub-saturating antibody concentrations do not change the result and can improve population resolution by reducing non-primary detector spillover. SSM values are indicative of fluorescence spectrum interactions and provide critical information for the prediction of immunofluorescence panel performance, the development of new panels and to provide additional information for determining the amount of antibody in multicolour panels [26]. In our study, 43 antibodies were titrated and the SSM values of seven panels were measured, providing information used to tweak the amount of antibody in the panels for the best assessment of the data.

In the innate immune system, the monocyte-macrophage lineage plays an important role in transplantation rejection [9]. After transplantation, monocytes can proliferate and differentiate into macrophages and DCs [9]. Different NK cell subsets are found to be associated with both kidney transplant rejection [59] as well as liver transplant tolerance [60]. Recent studies in bone marrow transplant recipients have shown that some NK cell subsets require a kind of education by T cells [61], and CD56+(bright)CD16+, CD56+(dim)CD16+ and CD56+(bright)CD16- NK cells are not only phenotypically but are functionally and developmentally different [28]. Neutrophils, the most abundant type of granulocytes, are a marker of transplant injury and there is now new evidence to suggest that neutrophil subsets have specialized effector functions and some subsets may be important for the promotion and maintenance of tolerance [10]. In addition to neutrophil subset numbers it is possible to assess the proportion expressing activation markers such as C5aR which is one of the factors responsible for neutrophil adhesion to endothelial cells [53, 54]. Using the panels described here, it is possible to measure both the proportion and number of several of these subsets from the monocyte-macrophage lineage simultaneously. For instance, not only were classical, intermediate, non-classical, and CD16+CD64+ monocyte assessed, but also CD16-CD56- monocytes [27], as well as CD16-CD56+ subsets which have been shown to expand in conditions associated with autoimmunity [62].

The current immune panel has the potential to allow us to better understand the impact of immunosuppression on leukocyte subsets. Lymphocyte depleting agents such as ATG create an environment for immune reconstitution that may be significantly different to what was seen pre-treatment. This affects not only T cell and B cell populations but other leukocyte populations. For instance, the Janus kinase inhibitor, tofacitinib causes a 50% reduction in NK cells and an absolute increase in CD19+B cells in renal transplant recipients [63]. Not only do T cells play a central role in both the effector and regulatory phases of rejection [11], FOXP3+Tregs suppress immune-cell proliferation and can potentially promote transplant tolerance [12–17]. TCRγδ T cells are classical actors of the innate immune response and are also suggested as a valuable immunological biomarker in the management of cytomegalovirus (CMV) infection after transplantation [64]. The ability to measure the number of memory-T cells may be important in the long term monitoring of transplant patients given their implication in chronic graft-rejection and their relative resistance to lymphocyte depleting agents [65]. In transplantation, the ability to identify subsets of Tregs, such as the alloantigen specific memory-Tregs may prove useful tools for monitoring the patient’s immune profile after immunomodulatory therapy [56]. In this study, we evaluated T cell activation by assessing the expression of CD57, HLA-DR, CD25, CXCR3, CD137 and CD154, and loss of CD27 or CD28. As well as assessing the dynamic T cell profile of naïve, central memory, effector memory and TEMRA by CD45RA/CD45RO and CCR7/CD62L [45–50, 52, 65]. The proportion and subsets of T cells based on TCRαβ and TCR γδ, or CD4 and CD8 expression was also assessed [64, 66]. The expression of CCR7/CD62L was evaluated on naïve (CD45RA+) and effector/memory (CD45RA-) CD25+CD127- Tregs. Furthermore, CD127, CD39, CD25, CD137 and CD154 were assessed on naïve (CD45RO-) and effector/memory (CD45RO+) FOXP3+ and FOXP3+bright Tregs for identification of a possible subset of alloantigen specific memory Tregs.

Host DCs are typical antigen presenting cells (APCs) that activate T cells by presenting donor alloantigenic peptides in the context of MHC, while tolerogenic DCs have been shown to promote antigen specific tolerance in animal models [67]. mDC1, mDC2, and PDCs were found to be decreased in PB of kidney transplant recipients on long-term immunosuppression [68]. In our study, we have been able to clearly identify and quantitate mDC1 and mDC2, PDCs, CD16+DCs and HLA-DR+ immature cells in order to correlate these with transplant outcome.

Recently, the role of B cells in transplantation have attracted more attention. B cells are involved in acute and chronic allograft-rejection by producing donor specific antibody and can impact the alloimmmune response as APCs [20]. In our study, CD19+B cells were assessed for the proportion of naïve and memory B cells, non-switched memory, class switched memory, transitional B cells and plasmablasts. Currently, we do not have definitive cell surface markers for Bregs [69]. In transplantation, the increase of the IgM+IgD+CD27- naïve or CD24hiCD38h transitional B cells in patients with stable grafts has been reported [20, 70]. In our study, possible Bregs (CD19+IgM+brightCD24+brightCD21+CD27-) were assessed and could be potentially used for comparison with naïve (CD19+CD27-IgD+) and transitional B cells (CD19+IgM+CD38+brightCD24+bright) in the transplant patients and correlated with outcome.

In conclusion, we have standardized the operating procedures, instrument setup and data analysis for absolute immune-cell-count and seven leukocyte multicolour panels using 12 parameters of a 5 laser flow cytometer. The settings allow for clear identification of multiple leukocyte subsets even when they are present in low frequency in peripheral blood. The results are reproducible and consistent over a 26-month period. Once established, the test is straight forward and easy to perform and requires only 1.5ml of WPB. We have demonstrated its utility in making longitudinal evaluations in islet transplant patients and it has the potential for monitoring transplant patients and other patients on long term immunosuppression and for determining if any changes in leukocyte subsets is associated with outcomes.

## Supporting information

S1 Fig Absolute cell-count panel. **(A)** The gating strategies for the absolute cell-count panel (panel 1). The first gate (G1) on time vs CD45 excludes poor flow and the second gate (G2) was for bead number on CD45 vs CD3. The third gate (G3) was for granulocytes and the fourth gate (G4) included monocytes, DCs and lymphocytes on CD45 vs SSC-A. The CD45 vs CD19, CD45 vs CD3, and CD45 vs CD14 showed CD19+B cells, CD3+T cells and CD14+monocytes respectively. The fifth gate (G5) for CD45+CD19- and the sixth gate (G6) for CD45+CD14- excluded B cells and monocytes, the CD56 vs CD3 identified CD56+CD3- NK cells and CD3+CD56+ NKT cells. The calculation formula for absolute cell-number is in the Methods. **(B)** Variation in the levels of absolute cell numbers of granulocytes, monocytes, B cells, T cells, NK cells, and NKT cells explained by differences between the five repeated-tests for each sample, and the subject measured, healthy control C2, C4 and C5.
(TIF)

S2 Fig Longitudinal calculation of CD3+ T cell numbers (CD4+, CD8+, CD4+CD8+ and CD4-CD8-) in 4 islet transplanted-recipients post-transplantation with ATG induction. Patient 1 received three islet transplants and the data showed 11 time points from pre-transplantation to 18 months after the 1^st^ islet transplantat (12 months 3^rd^ transplantation). Patient 2 received two islet transplants and the data showed at 9 time points from pre to 18 months after the 1^st^ islet transplantat (12 months 2^nd^ transplantation). Patients 4 and 10 received one islet transplant and the data shown is from pre-transplantation to 6 months after islet transplantation (5 time points for patient 4 and 4 time points for patient 10).
(TIF)

S3 Fig Measurement of the CD4/CD8 T cell ratio in 4 islet transplant-recipients post-transplantation with ATG induction. The percentage of CD4+, CD8+, CD4+CD8+, CD4-CD8- in CD3 T cells in patient 1 for from pre to18 months post-transplantation1^st^ islet transplant (12 months 3^rd^ transplantation), in patient 2 for 9 time points from pre to 18 months after the 1^st^ islet transplant (12 months 2^nd^ transplantation), and in patients 4 (5 time points) and 10 (4 time points) from pre-transplant to 6 months after islet transplantation showed a reversal of the CD4/CD8 T cell ratio post transplantation.
(TIF)

S4 Fig Detection of consistent B cell subsets pre and post transplantation over a 26 months period. The evaluation of B cell subsets after gating on CD19+ B cells, and assessing the CD27 vs IgD (panel 4 or B cell panel) from patient 2 (P2) pre-transplantation, 2 weeks, 1 and 3 months after the first islet transplant, and 1, 3, 6, 12 months after the second islet transplant across 26 months. The data showed that the four subsets of CD19+ B cells (CD27+IgD-, CD27-IgD+, CD27-IgD-, CD27+IgD+) were consistently detected with changes on population frequencies pre and post transplantation.
(TIF)

S5 Fig Comparison between 3 antibody clones for CD56. **(A)** The comparison of clones NCAM16.2, My31 and B519 of CD56-PE antibodies in panel 2. After gating on CD19- lymphocytes (G6b) in Fig 3A, the dot-plots of CD56 vs CD3 showed that separation of CD56+dim and CD56+bright cells was better using clone NCAM16.2, when compared to My31 and B519. The final concentrations were 0.31 μl/mL for NCAM16.2, and 0.25 μl /mL for My31 and B519 which were the antibody concentrations that gave the best staining index. **(B)** Fixation/permeabilization procedure impacted identification of CD25+CD127- Tregs using BV650-CD127 (HIL-7R-M21) in panel 8 (S3 Table). The proportion of CD25+CD127dim/- Tregs (gating on CD4+ T cells) decreased after fixation/permeabilization procedure and before the anti-FOXP3 antibody was added (5.6% with fixation/permeabilization v 8.1% without fixation/permeabilization).
(TIF)

S6 Fig The comparison of CD141 staining with 3 fluorochromes and 2 clones in panel 3. **(A),** The correlation between BV711-CD141 (1A4) and APC-CD141 (AD5-14H12). The staining pattern for BV711-CD141 vs V450-CD16, APC-CD141 vs V450-CD16, and BV711-CD141 vs APC-CD141 from the WPB control samples. The top row are panel 3 cocktail antibodies without anti-CD141 antibody and the second row are panel 3 cocktail antibodies with BV711-CD141 (1A4), and additional APC-CD141 (AD5-14H12). **B,** The results of the comparison of BV711-CD141 (1A4) and FITC-CD141 (AD5-14H12). The staining pattern for BV711-CD141 vs V450-CD16 from panel 3, and FITC-CD141 vs APC-H7-CD16 from panel 3 were assessed in three healthy-control samples.
(TIF)

S1 Table Additional tested antibodies. The clones and fluorochrome formats of 21 additional tested antibodies, including one lineage cocktail (CD3, CD14, CD19, CD20, CD56), are listed.
(PDF)

S2 Table Tested panels for general immune phenotype, DCs and T cell activation. The tested general immune phenotype panel (tested panel 2), one DCs panel (tested panel 3) and one T cell activation panel (tested panel 5) are listed. The fluorochrome formats for each antibody (clone) on the parameter (laser and filter) of the 5 laser 18 parameter BD-LSR Fortessa are also shown.
(PDF)

S3 Table Tested panels for naïve, memory and TCRαβ/TCRγδ T cells, and FOXP3+ Tregs. The two tested memory and naïve T cell panels (tested panel 6), TCRαβ/TCRγδ T cells, and state of T cells and neutrophils panel (tested panel 7), and tested FOXP3 Tregs panel (tested panel 8) are listed. The fluorochrome formats for each antibody (clone) on the parameter (laser and filter) of the 5 laser 18 parameter BD-LSR Fortessa are also shown.
(PDF)

S4 Table Spillover spreading matrix of panel 2. The SSM for the combination of fluorochromes used in panel 2 was calculated using FlowJo V10. The individual fluorochrome contributions to decreased sensitivity of other detectors are listed.
(PDF)

S5 Table Spillover spreading matrix of panel 3. The SSM for the combination of fluorochromes used in panel 3 was calculated using FlowJo V10. The individual fluorochrome contributions to decreased sensitivity of other detectors are listed.
(PDF)

S6 Table Spillover spreading matrix of panel 4. The SSM for the combination of fluorochromes used in panel 4 was calculated using FlowJo V10. The individual fluorochrome contributions to decreased sensitivity of other detectors are listed.
(PDF)

S7 Table Spillover spreading matrix of panel 5. The SSM for the combination of fluorochromes used in panel 5 was calculated using FlowJo V10. The individual fluorochrome contributions to decreased sensitivity of other detectors are listed.
(PDF)

S8 Table Spillover spreading matrix of panel 6. The SSM for the combination of fluorochromes used in panel 6 was calculated using FlowJo V10. The individual fluorochrome contributions to decreased sensitivity of other detectors are listed.
(PDF)

S9 Table Spillover spreading matrix of panel 7. The SSM for the combination of fluorochromes used in panel 7 was calculated using FlowJo V10. The individual fluorochrome contributions to decreased sensitivity of other detectors are listed.
(PDF)

S10 Table Spillover spreading matrix of panel 8. The SSM for the combination of fluorochromes used in panel 8 was calculated using FlowJo V10. The individual fluorochrome contributions to decreased sensitivity of other detectors are listed.
(PDF)

S11 Table Comparisons across panels. The population frequencies for CD3+ (panel 2, 5, 6, 7 and 8), CD4+ (panel 2, 5, 6, 7 and 8), CD16+ (panel 2, 3 and 7), CD27 (panel 4 and 7),and CD127+ (panel 6 and 8) in CD45+ cells were measured using different panels for the five healthy control and five pre-transplantation patients. Coefficient of variation (CV) of selected population frequencies was calculated across panels for controls (C1-C5, n = 5) and patients at pre-transplantation (P1-P5, n = 5). *CD45+ cells executing granulocytes. †Population frequency (%) of CD45+ cells. ‡C: Health control. ** P: Patient.
(PDF)

S12 Table Test of technical repeatability. Whole-peripheral-blood (WPB) samples from three healthy controls (C) were collected. An aliquot 50μl-WPB of each test in Trucount tube for five repeated-tests (Panel 1) was stained as staining protocol 1. Absolute cell number of granulocytes, monocytes, B cell, T Cell, NK and NKT cells per 1 μl of blood are shown. Coefficient of variation (CV) of immune cell counts was calculated across 5 repeated tests for each control sample on Control2, C4 and C5.
(PDF)

S13 Table Frequency of selected T subsets over time and their CVs. The CD4+ and CD8+ population frequencies in CD3 T cells in panel 2, 5 and 7, and CD25+CD127- Treg population frequencies in CD4 T cells in panel 6 and 8 in three heath controls over time. *Whole-peripheral-blood samples of heath control (C) 2, C4, C6 were taken at three or two separate time points. †Coefficient of variation (CV) of CD4+ and CD8+ population frequencies (% of CD3+ T cells) and CD25+CD127- Tregs (% of CD4+ T cells) over time was calculated on panel 2, 5 and 7 for CD4+ and CD8+ T cells, and panel 6 and 8 for CD25+CD127- Tregs.
(PDF)

S14 Table Frequency of low-numbered cell populations over time. The frequency of HLA-DR+CD45RA- T cells in CD3+CD4+ T cells (panel 5), TCRγδ T cells in CD45+CD3+ T cells (panel 7), CD127+CD45RO+ Tregs in CD4+FOXP3+ Tregs (panel 8), CD56+brightCD16- NK cells in NK cells (panel 2), CD56+CD16- monocytes in CD14+ monocytes (panel 2), mDC2 (CD141+mid/high DCs) in CD11c+HLA-R+ DCs (panel 3), CD27+IgD- memory B cells in CD45+CD19+ B cells (panel 4) in three heath controls over time. * Whole-peripheral-blood samples of heath control (C) 2, C4, C6 were taken at three or two separate time points. †The CV of these low-numbered cell population frequencies was calculated over time on different panels.
(PDF)
