# Supplementary figures and images for: Standardisation of flow cytometry for whole blood immunophenotyping of islet transplant and transplant clinical trial recipients
Source: PLoS One. 2019 May 22;14(5):e0217163. doi: 10.1371/journal.pone.0217163 (PMC6530858; doi:10.1371/journal.pone.0217163)

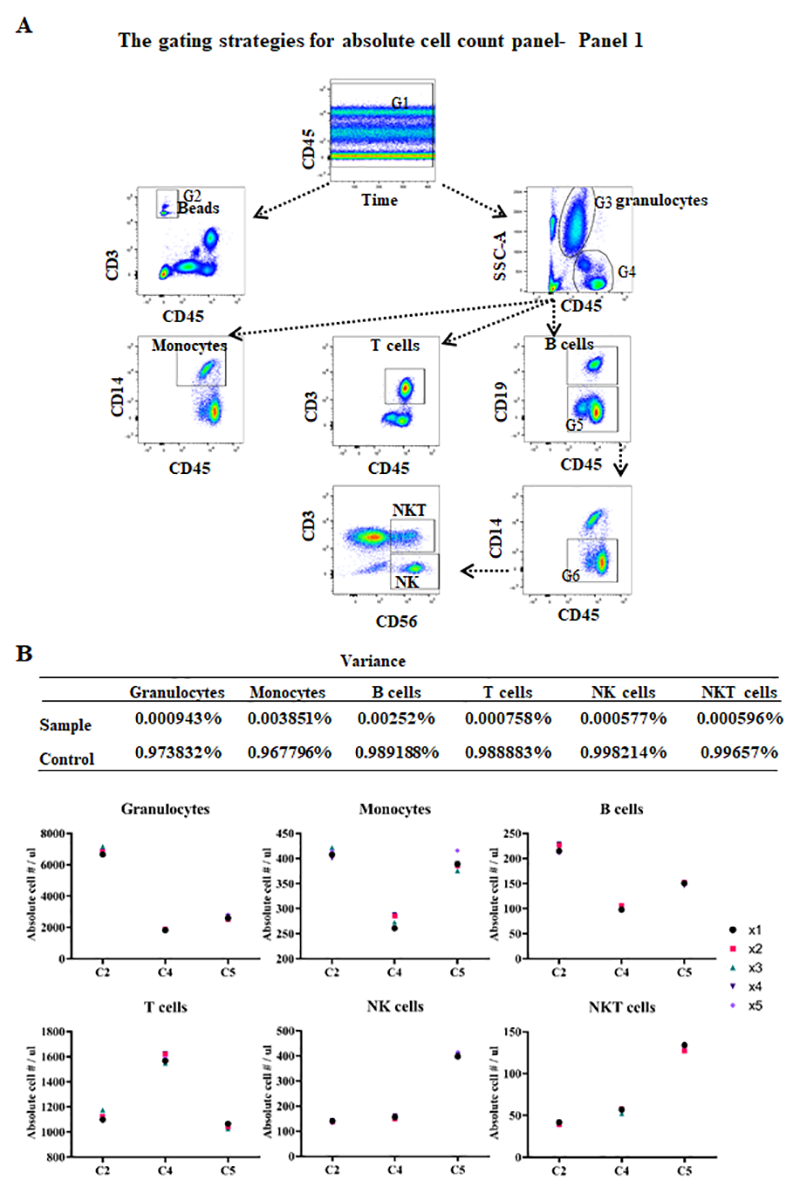

Supplement: S1 Fig — (A) The gating strategies for the absolute cell-count panel (panel 1). The first gate (G1) on time vs CD45 excludes poor flow and the second gate (G2) was for bead number on CD45 vs CD3. The third gate (G3) was for granulocytes and the fourth gate (G4) included monocytes, DCs and lymphocytes on CD45 vs SSC-A. The CD45 vs CD19, CD45 vs CD3, and CD45 vs CD14 showed CD19+B cells, CD3+T cells and CD14+monocytes respectively. The fifth gate (G5) for CD45+CD19- and the sixth gate (G6) for CD45+CD14- excluded B cells and monocytes, the CD56 vs CD3 identified CD56+CD3- NK cells and CD3+CD56+ NKT cells. The calculation formula for absolute cell-number is in the Methods. (B) Variation in the levels of absolute cell numbers of granulocytes, monocytes, B cells, T cells, NK cells, and NKT cells explained by differences between the five repeated-tests for each sample, and the subject measured, healthy control C2, C4 and C5. (TIF) [file pone.0217163.s001.tif]

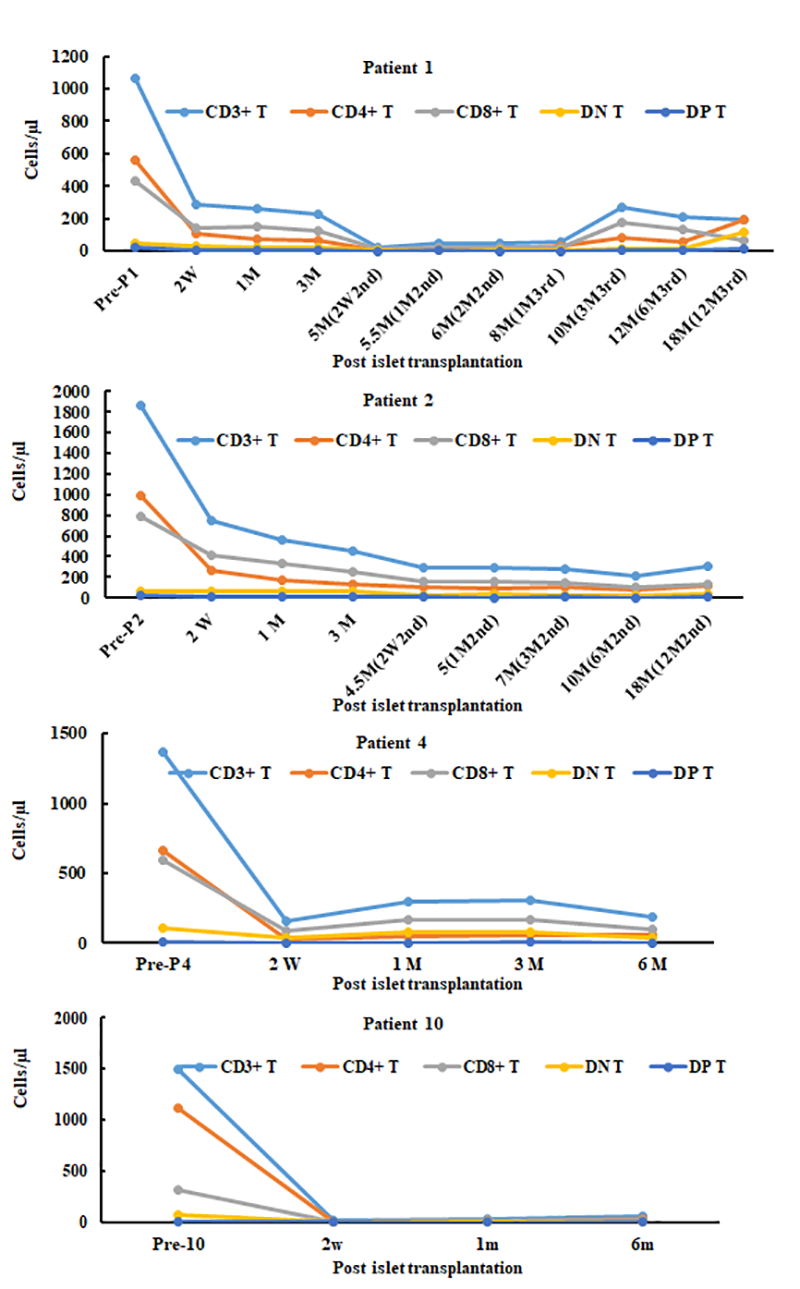

Supplement: S2 Fig — Patient 1 received three islet transplants and the data showed 11 time points from pre-transplantation to 18 months after the 1st islet transplantat (12 months 3rd transplantation). Patient 2 received two islet transplants and the data showed at 9 time points from pre to 18 months after the 1st islet transplantat (12 months 2nd transplantation). Patients 4 and 10 received one islet transplant and the data shown is from pre-transplantation to 6 months after islet transplantation (5 time points for patient 4 and 4 time points for patient 10). (TIF) [file pone.0217163.s002.tif]

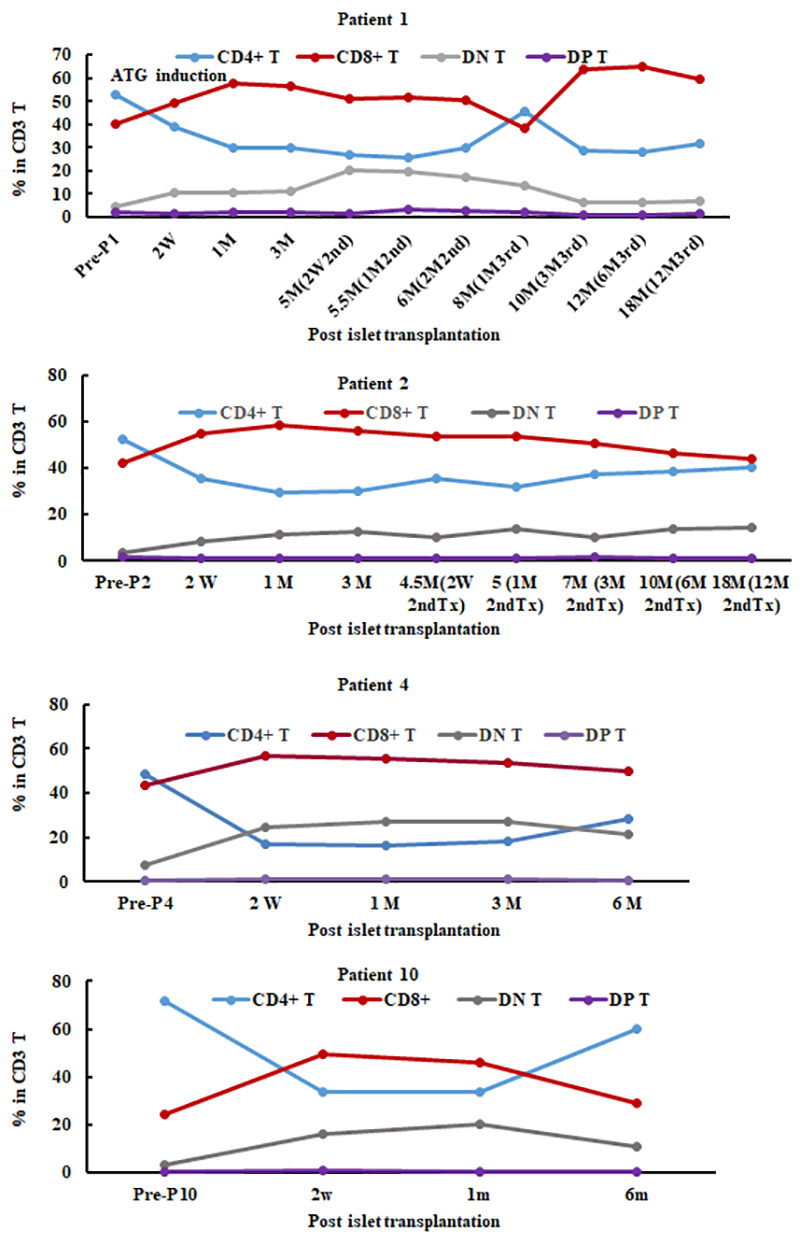

Supplement: S3 Fig — The percentage of CD4+, CD8+, CD4+CD8+, CD4-CD8- in CD3 T cells in patient 1 for from pre to18 months post-transplantation1st islet transplant (12 months 3rd transplantation), in patient 2 for 9 time points from pre to 18 months after the 1st islet transplant (12 months 2nd transplantation), and in patients 4 (5 time points) and 10 (4 time points) from pre-transplant to 6 months after islet transplantation showed a reversal of the CD4/CD8 T cell ratio post transplantation. (TIF) [file pone.0217163.s003.tif]

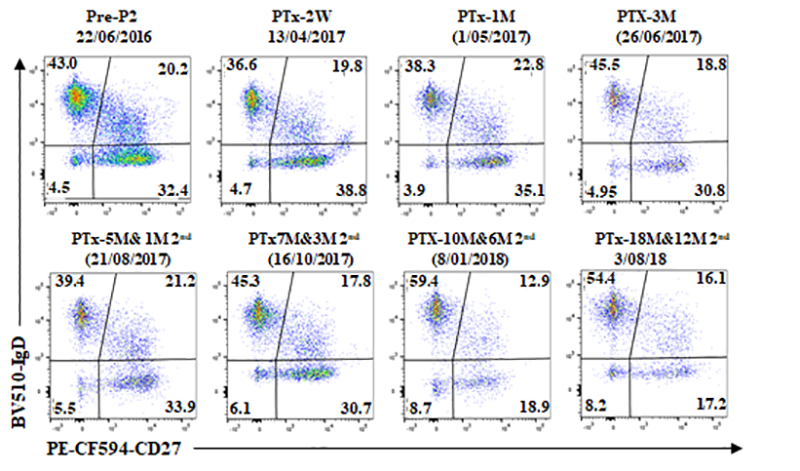

Supplement: S4 Fig — The evaluation of B cell subsets after gating on CD19+ B cells, and assessing the CD27 vs IgD (panel 4 or B cell panel) from patient 2 (P2) pre-transplantation, 2 weeks, 1 and 3 months after the first islet transplant, and 1, 3, 6, 12 months after the second islet transplant across 26 months. The data showed that the four subsets of CD19+ B cells (CD27+IgD-, CD27-IgD+, CD27-IgD-, CD27+IgD+) were consistently detected with changes on population frequencies pre and post transplantation. (TIF) [file pone.0217163.s004.tif]

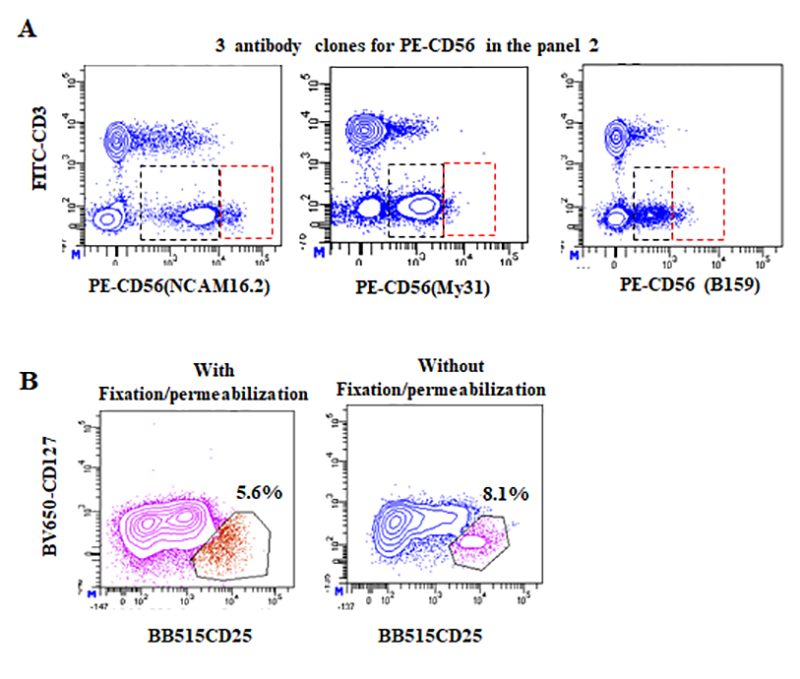

Supplement: S5 Fig — (A) The comparison of clones NCAM16.2, My31 and B519 of CD56-PE antibodies in panel 2. After gating on CD19- lymphocytes (G6b) in Fig 3A, the dot-plots of CD56 vs CD3 showed that separation of CD56+dim and CD56+bright cells was better using clone NCAM16.2, when compared to My31 and B519. The final concentrations were 0.31 μl/mL for NCAM16.2, and 0.25 μl /mL for My31 and B519 which were the antibody concentrations that gave the best staining index. (B) Fixation/permeabilization procedure impacted identification of CD25+CD127- Tregs using BV650-CD127 (HIL-7R-M21) in panel 8 (S3 Table). The proportion of CD25+CD127dim/- Tregs (gating on CD4+ T cells) decreased after fixation/permeabilization procedure and before the anti-FOXP3 antibody was added (5.6% with fixation/permeabilization v 8.1% without fixation/permeabilization). (TIF) [file pone.0217163.s005.tif]

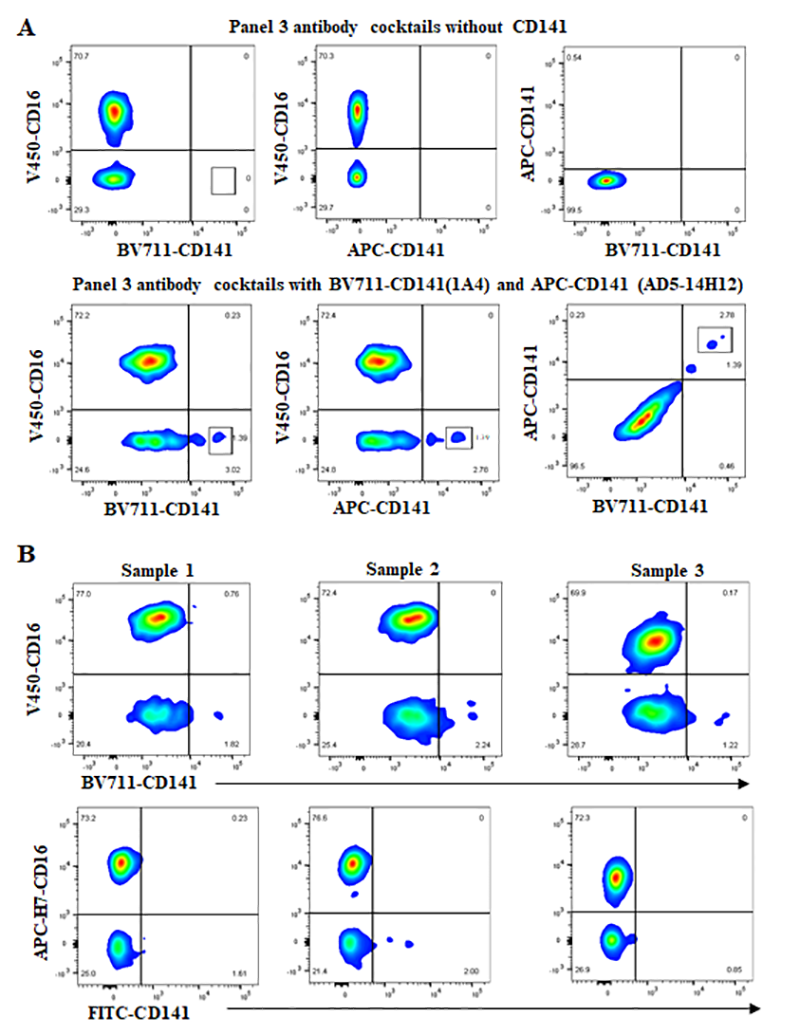

Supplement: S6 Fig — (A), The correlation between BV711-CD141 (1A4) and APC-CD141 (AD5-14H12). The staining pattern for BV711-CD141 vs V450-CD16, APC-CD141 vs V450-CD16, and BV711-CD141 vs APC-CD141 from the WPB control samples. The top row are panel 3 cocktail antibodies without anti-CD141 antibody and the second row are panel 3 cocktail antibodies with BV711-CD141 (1A4), and additional APC-CD141 (AD5-14H12). B, The results of the comparison of BV711-CD141 (1A4) and FITC-CD141 (AD5-14H12). The staining pattern for BV711-CD141 vs V450-CD16 from panel 3, and FITC-CD141 vs APC-H7-CD16 from panel 3 were assessed in three healthy-control samples. (TIF) [file pone.0217163.s006.tif]
