# Supplementary material for: Standardisation of flow cytometry for whole blood immunophenotyping of islet transplant and transplant clinical trial recipients
Source: PLoS One. 2019 May 22;14(5):e0217163. doi: 10.1371/journal.pone.0217163 (PMC6530858; doi:10.1371/journal.pone.0217163)
Supplement: S1 Table — The clones and fluorochrome formats of 21 additional tested antibodies, including one lineage cocktail (CD3, CD14, CD19, CD20, CD56), are listed. (PDF) [file pone.0217163.s007.pdf]

S1 Table. Additional tested antibodies

Antibody	Clone	Format
CD3	SK7	APC-H7
CD4	RPA-T4	APC
CD8	RPA-T8	AF-700
CD8	RPA-T8	APC-H7
CD11c	B-ly6	PE-cy7
CD16	3G8	APC-H7
CD28	CD28.2	BUV737
CD45	J.33	Krome Orange
CD45RO	UCHL1	BV711
CD56	B159	PE
CD56	My31	PE
CD62L	DREG-56	PE
CD62L	DREG-57	BV650
CD64	10.1	Pacific Blue
CD64	10.1	APC-R700
CD127	HIL-7R-M21	BV650
CD141(BDCA3)	AD5-14H12	APC
CD141(BDCA3)	AD5-14H12	FITC
CD197(CCR7)	3D12	BV711
HLA-DR	G46-6	V450
ILN (CD3, CD14<br>(CD19, CD20<br>CD56	SK7, SJ25C1,<br>NCAM16.2<br>L27, MφP9	PE
