# Supplementary material for: Standardisation of flow cytometry for whole blood immunophenotyping of islet transplant and transplant clinical trial recipients
Source: PLoS One. 2019 May 22;14(5):e0217163. doi: 10.1371/journal.pone.0217163 (PMC6530858; doi:10.1371/journal.pone.0217163)
Supplement: S2 Table — The tested general immune phenotype panel (tested panel 2), one DCs panel (tested panel 3) and one T cell activation panel (tested panel 5) are listed. The fluorochrome formats for each antibody (clone) on the parameter (laser and filter) of the 5 laser 18 parameter BD-LSR Fortessa are also shown. (PDF) [file pone.0217163.s008.pdf]

S2 Table. Tested panels for general immune phenotype, DCs and T cell activation

**Laser nm**	**355**		**403**			**488**	**561**			**639**		
**Laser mw**	**20**		**50**			**50-100**	**50**			**40**		
**Filter**	**379/28**	**740/28**	**440/40**	**525/50**	**710/50**	**488/10**	**586/15**	**610/20**	**780/60**	**670/14**	**730/45**	**730/45**
Tested Panel 2 - General immune phenotype-1												
CD			64	45		16	56	19	14	4	8	3
Clone			10.1	J.33		3G8	B159	HIB19	M5E2	RPA-T4	RPA-T8	SK7
Format			Pacific Blue	Krome Orange		FITC	PE	PE-CF594	PE-Cy7	APC	AF-700	APC-H7
Tested Panel 2 - General immune phenotype-2												
CD	45		16			3	56	19	14	4	64	8
Clone	H130		3G8			SK7	B159	HIB19	M5E2	RPA-T4	10.1	RPA-T8
Format	BUV395		V450			FITC	PE	PE-CF594	PE-Cy7	APC	APC-R700	APC-H7
Tested Panel 3 - DCs												
CD			HLA-DR	45		141	LIN	CD123	CD11c	303		CD16
Clone			G46-6	J.33		AD5-14H12		7G3	B-ly6	AC144		3G8
Format			V450	Krome Orange		FITC	PE	PE-CF594	PE-cy7	APC		APC-H7
Tested Panel 5 - T cell activation												
CD	45	28	4	HLA-DR	27	3		57	45RA	8		
Clone	H130	CD28.2	RPA-T4	G46-6	L128	SKY7		NK-1	L48	RPA-T8		
Format	BUV395	BUV737	V450	BV510	BV711	FITC		PE-CF594	PE-cy7	APC		
