# Supplementary material for: Standardisation of flow cytometry for whole blood immunophenotyping of islet transplant and transplant clinical trial recipients
Source: PLoS One. 2019 May 22;14(5):e0217163. doi: 10.1371/journal.pone.0217163 (PMC6530858; doi:10.1371/journal.pone.0217163)
Supplement: S3 Table — The two tested memory and naïve T cell panels (tested panel 6), TCRαβ/TCRγδ T cells, and state of T cells and neutrophils panel (tested panel 7), and tested FOXP3 Tregs panel (tested panel 8) are listed. The fluorochrome formats for each antibody (clone) on the parameter (laser and filter) of the 5 laser 18 parameter BD-LSR Fortessa are also shown. (PDF) [file pone.0217163.s009.pdf]

S3 Table. Tested panels for naïve, memory and TCRαβ/TCRγδ T cells, and FOXP3+ Tregs

**Laser nm**	**355**		**403**				**488**	**561**			**639**	
**Laser mw**	**20**		**50**				**50-100**	**50**			**40**	
**Filter**	**379/28**	**740/35**	**440/40**	**525/50**	**660/20**	**710/50**	**488/10**	**586/15**	**610/20**	**780/60**	**670/14**	**780/60**
Tested Panel 6 - Memory and Naïve T cells-1												
CD	45		4	3		197	25	62L	127	45RA		8
Clone	H130		RPA-T4	UCHT1		3D12	2A3	DREG-56	HIL-7R-M21	HI100		RPA-T8
Format	BUV395		V450	BV510		BV711	BB515	PE	PE-CF594	PE-Cy7		APC-H7
Tested Panel 6 - Memory and Naïve T cells-2												
CD	45		4	3	62L		25	197	127	45RA	8	
Clone	H130		RPA-T4	UCHT1	DREG-56		2A3	150503	HIL-7R-M21	HI100	RPA-T8	
Format	BUV395		V450	BV510	BV650		BB515	PE	PE-CF594	PE-Cy7	APC	
Tested Panel 7 - TCRαβ/TCRγδ & T cell state & neutrophils												
CD	45	TCRαβ	4	3		45RO	16	88	183	TCRγδ	8	
Clone	H130	T10B9.1A-31	RPA-T4	UCHT1		UCHL1	NKP15	C85-4124	1C6	11F2	RPA-T8	
Format	BUV395	BUV737	V450	BV510		BV711	FITC	PE	PE-CF594	PE-Cy7	APC	
Tested Panel 8 – FOXP3 Tregs -1												
CD	45	39	137	3	127	45RO	25		FOXP3	4	154	
Clone	H130	TU66	4B4-1	UCHT1	HIL-7R-M21	UCHL1	2A3		259D/C7	RPA-T4	TRAP-1	
Format	BUV395	BUV737	V450	BV510	BV650	BV711	BB515		PE-CF594	PE-Cy7	APC	
