# Supplementary material for: Standardisation of flow cytometry for whole blood immunophenotyping of islet transplant and transplant clinical trial recipients
Source: PLoS One. 2019 May 22;14(5):e0217163. doi: 10.1371/journal.pone.0217163 (PMC6530858; doi:10.1371/journal.pone.0217163)
Supplement: S4 Table — The SSM for the combination of fluorochromes used in panel 2 was calculated using FlowJo V10. The individual fluorochrome contributions to decreased sensitivity of other detectors are listed. (PDF) [file pone.0217163.s010.pdf]

S4 Table. Spillover spreading matrix of the Panel 2

Panel 2	FITC CD3	APC CD8	APC-R700 CD64	BUV395 CD45	V450 CD16	V500 CD4	PE CD56	PE-CF594 CD19	PE-Cy7 CD14	Sum
**FITC CD3**	0	0.0451	0	0.0611	0	0.884	0	0	0.0304	1.0206
**APC CD8**	0.0237	0	0.761	0	0	0.0638	0.0512	0.126	1.07	2.0957
**APC-R700 CD64**	0.0525	1.2	0	0	0	0	0.0556	0.0662	3.01	4.3843
**BUV395 CD45**	0	0.0519	0	0	0.0987	0	0	0	0	0.1506
**V450 CD16**	0.0216	0.027	0.0189	0	0	0.697	0.0369	0.0018	0.0262	0.8294
**V500 CD4**	0.15	0	0.0179	0	0.212	0	0	0	0	0.3799
**PE CD56**	0.0797	0.0173	0.0075	0.0257	0	0.0782	0	1.24	0.287	1.7354
**PE-CF594 CD19**	0.031	0.103	0.0312	0	0.0255	0.013	1.21	0	0.838	2.2517
**PE-Cy7 CD14**	0.0307	0.0366	0.0758	0	0	0	0.299	0.166	0	0.6081
**Sum**	0.3892	1.4809	0.9123	0.0868	0.3362	1.736	1.6527	1.6	5.2616	
