# Supplementary material for: Standardisation of flow cytometry for whole blood immunophenotyping of islet transplant and transplant clinical trial recipients
Source: PLoS One. 2019 May 22;14(5):e0217163. doi: 10.1371/journal.pone.0217163 (PMC6530858; doi:10.1371/journal.pone.0217163)
Supplement: S5 Table — The SSM for the combination of fluorochromes used in panel 3 was calculated using FlowJo V10. The individual fluorochrome contributions to decreased sensitivity of other detectors are listed. (PDF) [file pone.0217163.s011.pdf]

S5 Table. Spillover spreading matrix of the Panel 3

Panel3	FITC LIN	APC CD303	BUV395 CD45	V450 CD16	BV510 HLA-DR	BV711 CD141	PE CD11c	PE-CF594 CD123	Sum
**FITC LIN**	0	0.0747	0.108	0	0.805	0	0	0	0.9877
**APC CD303**	0.035	0	0.0468	0	0.0358	0.965	0.0386	0.168	1.2892
**BUV395 CD45**	0.043	0.0295	0	0.277	0.107	0.0213	0.037	0	0.5148
**V450 CD16**	0.036	0.0022	0	0	0.697	0.0794	0	0	0.8146
**BV510 HLA-DR**	0.1	0.0335	0	0.371	0	0.687	0.0318	0.0367	1.26
**BV711 CD141**	0	0.231	0.0581	0.592	0.177	0	0.0337	0.0493	1.1411
**PE CD11c**	0.07	0.015	0	0.0209	0.0613	0.233	0	1.4	1.8002
**PE-CF594 CD123**	0.028	0.082	0	0.0472	0	0.819	1.34	0	2.3162
**Sum**	0.314	0.469	0.2129	1.3081	1.8831	2.8047	1.4811	1.654	
