# Supplementary material for: Standardisation of flow cytometry for whole blood immunophenotyping of islet transplant and transplant clinical trial recipients
Source: PLoS One. 2019 May 22;14(5):e0217163. doi: 10.1371/journal.pone.0217163 (PMC6530858; doi:10.1371/journal.pone.0217163)
Supplement: S6 Table — The SSM for the combination of fluorochromes used in panel 4 was calculated using FlowJo V10. The individual fluorochrome contributions to decreased sensitivity of other detectors are listed. (PDF) [file pone.0217163.s012.pdf]

S6 Table. Spillover spreading matrix of the Panel 4

Panel 4	BB515 CD19	APC IgM	BUV395 CD45	V421 CD38	BV510 IgD	PE CD21	PE-CF594 CD27	PE-Cy7 CD24	Sum
**BB515 CD19**	0	0.041	0	0.0201	0.428	0	0	0	0.4891
**APC IgM**	0	0	0	0.154	0.468	0.091	0.128	0.908	1.749
**BUV395 CD45**	0	0.056	0	0.0751	0	0	0	0.0307	0.1618
**V421 CD38**	0.0487	0	0	0	0.711	0.0308	0.0141	0.0276	0.8322
**BV510 IgD**	0.107	0.0317	0	0.1	0	0	0.0272	0	0.2659
**PE CD21**	0.0645	0.0228	0.0402	0	0.0672	0	1.21	0.25	1.6547
**PE-CF594 CD27**	0.031	0.109	0	0	0	1.19	0	0.785	2.115
**PE-Cy7 CD24**	0.0264	0.0434	0	0.0193	0	0.355	0.197	0	0.6411
**Sum**	0.2776	0.3039	0.0402	0.3685	1.6742	1.6668	1.5763	2.0013	
