# Supplementary material for: Standardisation of flow cytometry for whole blood immunophenotyping of islet transplant and transplant clinical trial recipients
Source: PLoS One. 2019 May 22;14(5):e0217163. doi: 10.1371/journal.pone.0217163 (PMC6530858; doi:10.1371/journal.pone.0217163)
Supplement: S7 Table — The SSM for the combination of fluorochromes used in panel 5 was calculated using FlowJo V10. The individual fluorochrome contributions to decreased sensitivity of other detectors are listed. (PDF) [file pone.0217163.s013.pdf]

S7 Table. Spillover spreading matrix of the Panel 5

**Panel 5**	BB515 CD8	APC CD28	BUV395 CD45	BUV740 CD3	V450 CD4	BV510 HLA-DR	BV711 CD27	PE-CF594 CD57	PE-Cy7 CDRA	Sum
**BB515 CD8**	0	0	0	0	0	0.461	0	0	0	0.461
**APC CD28**	0.0893	0	0.0776	0.955	0	0.0952	1.05	0.121	0.834	3.2221
**BUV395 CD45**	0	0.0705	0	0.2	0.193	0.144	0	0.103	0.0794	0.7899
**BUV740 CD3**	0.0617	0.135	0.258	0	0	0	1.2	0.0617	0.579	2.2954
**V450 CD4**	0.0286	0	0	0	0	0.42	0.0689	0.0286	0.0286	0.5747
**BV510 HLA-DR**	0.103	0	0	0.505	0.314	0	0.701	0	0.0558	1.6788
**BV711 CD27**	0	0.239	0	2.95	0.446	0.12	0	0	0.345	4.1
**PE-CF594 CD57**	0.0333	0.104	0	0.407	0	0	0.839	0	0.826	2.2093
**PE-Cy7 CDRA**	0.0398	0.0398	0	0.432	0	0	0.0925	0.181	0	0.7851
**Sum**	0.3557	0.5883	0.3356	5.449	0.953	1.2402	3.9514	0.4953	2.7478	
