# Supplementary material for: Standardisation of flow cytometry for whole blood immunophenotyping of islet transplant and transplant clinical trial recipients
Source: PLoS One. 2019 May 22;14(5):e0217163. doi: 10.1371/journal.pone.0217163 (PMC6530858; doi:10.1371/journal.pone.0217163)
Supplement: S8 Table — The SSM for the combination of fluorochromes used in panel 6 was calculated using FlowJo V10. The individual fluorochrome contributions to decreased sensitivity of other detectors are listed. (PDF) [file pone.0217163.s014.pdf]

S8 Table. Spillover spreading matrix of the Panel 6

Panel 6	BB515 CD25	APC CD8	BUV395 CD45	V450 CD4	BV510 CD3	BV711 CD62L	PE CCR7	PE-CF594 CD127	PE-Cy7 CDRA	Sum
BB515 CD25	0	0.0181	0	0.0456	0.414	0.0256	0	0	0	0.5033
APC CD8	0.0055	0	0.0408	0.0814	0	1.05	0.0429	0.131	0.838	2.1896
BUV395 CD45	0	0	0	0.182	0	0	0	0	0	0.182
V450 CD4	0.0224	0	0.0422	0	0.704	0.1	0	0	0	0.8686
BV510 CD3	0.088	0.0268	0	0.362	0	0.706	0	0.03	0.0158	1.2286
BV711 CD62L	0.0274	0.182	0.0531	0.437	0.0725	0	0	0	0.342	1.114
PE CCR7	0.0702	0.0253	0	0	0.0601	0.263	0	1.21	0.298	1.9266
PE-CF594 CD127	0.0332	0.0877	0	0.0487	0	0.82	1.2	0	0.884	3.0736
PE-Cy7 CDRA	0.0156	0.0322	0	0	0	0.0826	0.289	0.158	0	0.5774
Sum	0.2623	0.3721	0.1361	1.1567	1.2506	3.0472	1.5319	1.529	2.3778	
