# Supplementary material for: Standardisation of flow cytometry for whole blood immunophenotyping of islet transplant and transplant clinical trial recipients
Source: PLoS One. 2019 May 22;14(5):e0217163. doi: 10.1371/journal.pone.0217163 (PMC6530858; doi:10.1371/journal.pone.0217163)
Supplement: S9 Table — The SSM for the combination of fluorochromes used in panel 7 was calculated using FlowJo V10. The individual fluorochrome contributions to decreased sensitivity of other detectors are listed. (PDF) [file pone.0217163.s015.pdf]

S9 Table. Spillover spreading matrix of the Panel 7

Panel 7	FITC CD16	APC CD8	BUV395 CD45	BUV737 TCRαβ	V450 CD4	BV510 CD3	BV786 CD45RO	PE C5aR	PE-CF594 CD193	PE-Cy TCRγδ	Sum
FITC CD16	0	0.0488	0	0.0969	0	0.896	0.104	0	0	0	1.1457
APC CD8	0.0312	0	0.0592	0.973	0.0738	0	0.386	0.0933	0.122	0.845	2.5835
BUV395 CD45	0.0711	0	0	0.232	0.198	0	0.106	0.0864	0	0	0.6935
BUV737 TCRαβ	0.0232	0.146	0.26	0	0.113	0	1.2	0.0854	0.0544	0.606	2.488
V450 CD4	0	0.0511	0	0.0064	0	0.668	0	0	0	0	0.7255
BV510 CD3	0.0826	0.0022	0	0.488	0.331	0	0.375	0	0.0157	0	1.2945
BV786 CD45RO	0	0.097	0	1.12	0.982	0.224	0	0.0306	0.0035	0.365	2.8221
PE C5aR	0.063	0.0019	0	0.164	0	0.0654	0.0977	0	1.25	0.306	1.948
PE-CF594 CD193	0.0305	0.1	0	0.402	0.0346	0.0303	0.279	1.24	0	0.782	2.8984
PE-Cy TCRγδ	0.036	0.0356	0	0.442	0.155	0	1.59	0.385	0.212	0	2.8556
Sum	0.3376	0.4826	0.3192	3.9243	1.8874	1.8837	4.1377	1.9207	1.6576	2.904	
