# Supplementary material for: Standardisation of flow cytometry for whole blood immunophenotyping of islet transplant and transplant clinical trial recipients
Source: PLoS One. 2019 May 22;14(5):e0217163. doi: 10.1371/journal.pone.0217163 (PMC6530858; doi:10.1371/journal.pone.0217163)
Supplement: S10 Table — The SSM for the combination of fluorochromes used in panel 8 was calculated using FlowJo V10. The individual fluorochrome contributions to decreased sensitivity of other detectors are listed. (PDF) [file pone.0217163.s016.pdf]

S10 Table. Spillover spreading matrix of the Panel 8

Panel 8	BB515 CD25	APC CD154	BUV395 CD45	BUV737 CD39	V421 CD137	BV510 CD3	BV786 CD45RO	PE CD127	PE-CF594 FOXP3	PE-Cy7 CD4	Sum
BB515 CD25	0	0	0	0	0	0.461	0	0	0	0	0.461
APC CD154	0.0893	0	0.0776	0.907	0	0.0952	0.442	0	0.121	0.854	2.5861
BUV395 CD45	0	0.0699	0	0.204	0.0975	0	0	0	0	0.0786	0.45
BUV737 CD39	0.0617	0.135	0.258	0	0.0874	0	1.24	0.0764	0.0616	0.595	2.5151
V421 CD137	0.0286	0	0	0	0	0.42	0.0338	0	0	0.0286	0.511
BV510 CD3	0.103	0	0	0.5	0.314	0	0.377	0	0	0	1.294
BV786 CD45RO	0.0005	0.11	0	1.18	0.935	0.237	0	0.0741	0.0637	0.371	2.9713
PE CD127	0.0733	0.0335	0	0.143	0	0.0701	0.108	0	1.34	0.288	2.0559
PE-CF594 FOXP3	0.0333	0.104	0	0.38	0	0	0.289	1.31	0	0.825	2.9413
PE-Cy7 CD4	0.0398	0.0398	0	0.432	0.145	0	1.55	0.382	0.199	0	2.7876
Sum	0.4295	0.4922	0.3356	3.746	1.5789	1.2833	4.0398	1.8425	1.7853	3.0402	
