# Supplementary material for: Standardisation of flow cytometry for whole blood immunophenotyping of islet transplant and transplant clinical trial recipients
Source: PLoS One. 2019 May 22;14(5):e0217163. doi: 10.1371/journal.pone.0217163 (PMC6530858; doi:10.1371/journal.pone.0217163)
Supplement: S11 Table — The population frequencies for CD3+ (panel 2, 5, 6, 7 and 8), CD4+ (panel 2, 5, 6, 7 and 8), CD16+ (panel 2, 3 and 7), CD27 (panel 4 and 7),and CD127+ (panel 6 and 8) in CD45+ cells were measured using different panels for the five healthy control and five pre-transplantation patients. Coefficient of variation (CV) of selected population frequencies was calculated across panels for controls (C1-C5, n = 5) and patients at pre-transplantation (P1-P5, n = 5). *CD45+ cells executing granulocytes. †Population frequency (%) of CD45+ cells. ‡C: Health control. ** P: Patient. (PDF) [file pone.0217163.s017.pdf]

S11 Table. Comparisons across panels

Type of CD45+*	Panel	Format/Clone	Population frequency %† (Control)					Population frequency % (Patient Pre)				
			C‡1	C2	C3	C4	C5	P**1	P2	P3	P4	P5
CD3+	2	FITC/SK7	60	59	40	64	55	43	55	57	55	65
	5	BUV737/UCHT1	60	59	39	63	54	44	56	57	54	64
	6	BV510/UCHT1	60	59	39	63	55	43	56	58	55	64
	7	BV510/UCHT1	59	58	40	62	54	44	56	58	55	64
	8	BV510/UCHT1	58	59	37	62	52	42	55	56	52	64
**Coefficient of variation**			**1.2**	**0.5**	**2.9**	**0.9**	**1.8**	**1.7**	**1**	**2**	**2.1**	**0.5**
CD4+	2	V500/RPA-T4	27	39	21	46	35	23	30	38	27	46
	5	V450/ RPA-T4	27	39	21	46	34	23	30	38	26	45
	6	V450/ RPA-T4	27	39	21	46	34	23	30	39	25	46
	7	V450/ RPA-T4	27	39	21	45	34	23	30	39	26	45
	8	PE-Cy7/ RPA-T4	27	39	19	45	33	22	30	37	25	45
**Coefficient of variation**			**1.5**	**0.3**	**3.5**	**1.3**	**1.9**	**2.6**	**0.7**	**1.7**	**2.9**	**1**
CD16+	2	V450/3G8	13	11	24	14	22	16	6.9	8.9	21	6.9
	3	V450/3G8	12	11	24	15	22	17	7.3	9.2	21	7
	7	FITC/ NKP15	13	11	25	14	21	16	7.1	8.9	20	6.7
**Coefficient of variation**			**1.2**	**0.5**	**1.7**	**1.7**	**2.2**	**0.9**	**2.8**	**2.1**	**2.2**	**2.2**
CD27+	4	PE-CF594/ M-T271	51	53	31	62	43	47	56	55	40	65
	7	BV711/ L128	52	53	30	60	42	50	56	54	39	63
**Coefficient of variation**			**1.8**	**0.7**	**3**	**1.9**	**2.2**	**3.9**	**1**	**1.2**	**0.7**	**2.1**
CD127+	6	PE-CF594/HIL.7R.M21	52	53	30	59	41	37	54	49	44	55
	8	PE/ HIL.7R.M21	50	53	28	60	42	35	52	47	45	55
**Coefficient of variation**			**2.1**	**0.5**	**4.4**	**1.8**	**1.9**	**2.5**	**2.4**	**2.8**	**1.2**	**0.2**
