# Supplementary material for: Standardisation of flow cytometry for whole blood immunophenotyping of islet transplant and transplant clinical trial recipients
Source: PLoS One. 2019 May 22;14(5):e0217163. doi: 10.1371/journal.pone.0217163 (PMC6530858; doi:10.1371/journal.pone.0217163)
Supplement: S12 Table — Whole-peripheral-blood (WPB) samples from three healthy controls (C) were collected. An aliquot 50μl-WPB of each test in Trucount tube for five repeated-tests (Panel 1) was stained as staining protocol 1. Absolute cell number of granulocytes, monocytes, B cell, T Cell, NK and NKT cells per 1 μl of blood are shown. Coefficient of variation (CV) of immune cell counts was calculated across 5 repeated tests for each control sample on Control2, C4 and C5. (PDF) [file pone.0217163.s018.pdf]

S12 Table. Test of technical repeatability

Control	Cell Type	1	2	3	4	5	CV
C2	Granulocytes	6660	6826	7169	6840	6669	3.0
	Monocytes	408	407	422	401	416	2.0
	B cells	215	227	230	229	211	3.9
	T cells	1099	1124	1175	1122	1102	2.7
	NK cells	141	138	141	144	133	2.9
	NKT cells	41.8	39.1	43	42.1	38.4	4.9
C4	Granulocytes	1831	1880	1809	1904	1853	2.0
	Monocytes	261	286	273	288	261	4.7
	B cells	98	106	98	100	105	3.9
	T cells	1568	1620	1547	1626	1595	2.1
	NK cells	157	150	149	161	160	3.6
	NKT cells	57	58	52	56	56	4.2
C5	Granulocytes	2595	2518	2704	2688	2825	4.3
	Monocytes	389	386	376	390	416	3.7
	B cells	151	153	151	146	154	2.0
	T cells	1066	1043	1026	1032	1068	1.8
	NK cells	398	398	407	405	415	1.7
	NKT cells	134	127	134	133	136	2.5
