# Supplementary material for: Standardisation of flow cytometry for whole blood immunophenotyping of islet transplant and transplant clinical trial recipients
Source: PLoS One. 2019 May 22;14(5):e0217163. doi: 10.1371/journal.pone.0217163 (PMC6530858; doi:10.1371/journal.pone.0217163)
Supplement: S13 Table — The CD4+ and CD8+ population frequencies in CD3 T cells in panel 2, 5 and 7, and CD25+CD127- Treg population frequencies in CD4 T cells in panel 6 and 8 in three heath controls over time. *Whole-peripheral-blood samples of heath control (C) 2, C4, C6 were taken at three or two separate time points. †Coefficient of variation (CV) of CD4+ and CD8+ population frequencies (% of CD3+ T cells) and CD25+CD127- Tregs (% of CD4+ T cells) over time was calculated on panel 2, 5 and 7 for CD4+ and CD8+ T cells, and panel 6 and 8 for CD25+CD127- Tregs. (PDF) [file pone.0217163.s019.pdf]

S13 Table. Frequency of selected T subsets over time and their CVs

Control*	Subsets/Cell Type (%)	Panel	1st	2nd	3rd	CV†
C2	CD4+/ CD3+ T	2	65.9	69.3	65.6	3.0
		5	66.3	69.5	65.3	3.2
		7	66.1	69.5	65.2	3.3
	CD8+/ CD3+ T	2	28.1	25.7	28.2	5.1
		5	27.7	25.2	27.5	5.1
		7	27.6	25.4	28.5	5.8
	CD25+CD127- / CD4+ T	6	5.67	5.82	5.89	1.9
		8	5.75	5.63	5.62	1.3
C4	CD4+/ CD3+ T	2	72.2	73.1		0.8
		5	71.8	72.6		0.8
		7	71.8	72.0		0.2
	CD8+/ CD3+ T	2	24.7	23.8		2.3
		5	24.7	25.3		1.7
		7	24.7	24.2		1.4
	CD25+CD127- / CD4+ T	6	6.50	6.85		3.7
		8	7.03	7.11		0.9
C6	CD4+/ CD3+ T	2	51.9	55.2		4.3
		5	50.9	54.2		4.4
		7	53.6	55.0		1.8
	CD8+/ CD3+ T	2	36.9	35.5		2.9
		5	37.0	35.1		3.7
		7	35.2	34.2		2.0
	CD25+CD127- / CD4+ T	6	7.45	7.90		4.1
		8	7.80	8.23		3.7
