# Supplementary material for: Standardisation of flow cytometry for whole blood immunophenotyping of islet transplant and transplant clinical trial recipients
Source: PLoS One. 2019 May 22;14(5):e0217163. doi: 10.1371/journal.pone.0217163 (PMC6530858; doi:10.1371/journal.pone.0217163)
Supplement: S14 Table — The frequency of HLA-DR+CD45RA- T cells in CD3+CD4+ T cells (panel 5), TCRγδ T cells in CD45+CD3+ T cells (panel 7), CD127+CD45RO+ Tregs in CD4+FOXP3+ Tregs (panel 8), CD56+brightCD16- NK cells in NK cells (panel 2), CD56+CD16- monocytes in CD14+ monocytes (panel 2), mDC2 (CD141+mid/high DCs) in CD11c+HLA-R+ DCs (panel 3), CD27+IgD- memory B cells in CD45+CD19+ B cells (panel 4) in three heath controls over time. * Whole-peripheral-blood samples of heath control (C) 2, C4, C6 were taken at three or two separate time points. †The CV of these low-numbered cell population frequencies was calculated over time on different panels. (PDF) [file pone.0217163.s020.pdf]

S14 Table. Frequency of low-numbered cell populations over time and their CVs

Control*	Subsets/ Cell Type (Percentage)	Panel	1st	2nd	3rd	CV†
C2	HLA-DR+CD45RA-/ CD3+CD4+ T	5	3.92	3.50	3.95	6.6
	TCR γδ/ CD45+CD3+ T	7	5.21	4.10	5.40	14.3
	CD127+CD45RO+ / FOXP3 Tregs	8	8.10	9.10	7.21	11.6
	CD56+brightCD16-/ NK cells	2	4.71	6.29	5.53	14.3
	CD56+CD16- / CD14+ monocytes	2	9.74	9.87	9.99	1.3
	mDC2/ CD11c+HLA-R+DCs	3	5.12	5.32	5.08	2.5
	CD27+IgD- memory/ CD45+CD19+ B	4	11.4	9.31	10.4	10.1
C4	HLA-DR+CD45RA-/ CD4+ T	5	5.80	6.13		3.9
	TCR γδ/ CD45+CD3+ T	7	1.10	0.90		14.1
	CD127+CD45RO+ / FOXP3 Tregs	8	15.9	15.6		1.3
	CD56+brightCD16-/ NK cells	2	2.94	3.36		9.4
	CD56+CD16- / CD14+ monocytes	2	13.3	12.3		5.5
	mDC2/ CD11c+HLA-R+DCs	3	4.52	4.18		5.5
	CD27+IgD- memory/ CD45+CD19+ B	4	21.6	18.4		11.3
C6	HLA-DR+CD45RA-/ CD4+ T	5	6.50	7.04		5.6
	TCR γδ/ CD45+CD3+ T	7	6.26	6.77		5.5
	CD127+CD45RO+ / FOXP3 Tregs	8	5.20	4.40		11.7
	CD56+brightCD16-/ NK cells	2	2.70	3.24		12.8
	CD56+CD16- / CD14+ monocytes	2	32.1	30.0		4.8
	mDC2/ CD11c+HLA-R+DCs	3	2.29	2.08		6.8
	CD27+IgD- memory/ CD45+CD19+ B	4	13.5	11.5		11.3
